# Effect of thromboelastography (TEG®) and rotational thromboelastometry (ROTEM®) on diagnosis of coagulopathy, transfusion guidance and mortality in trauma: descriptive systematic review

**DOI:** 10.1186/s13054-014-0518-9

**Published:** 2014-09-27

**Authors:** Luis Teodoro Da Luz, Bartolomeu Nascimento, Ajith Kumar Shankarakutty, Sandro Rizoli, Neill KJ Adhikari

**Affiliations:** Department of Critical Care Medicine, Sunnybrook Health Sciences Centre and University of Toronto, 2075 Bayview Avenue Room D1.08, Toronto, ON M4N 3M5 Canada; Department of Surgery, Sunnybrook Health Sciences Centre and University of Toronto, 2075 Bayview Avenue Room H1.89, Toronto, ON M4N 3M5 Canada; Departamento de Cirurgia e Anatomia, Faculdade de Medicina de Ribeirão Preto, Universidade de São Paulo, Av. Bandeirantes, 3900, 9° andar do Hospital das Clínicas de Ribeirão Preto, Ribeirão Preto, São Paulo, Brasil; Departments of Surgery and Critical Care Medicine, St. Michael’s Hospital and University of Toronto, 30 Bond Street, 3074 Donnelly Wing, Toronto, ON M5W 1B8 Canada

## Abstract

**Introduction:**

The understanding of coagulopathies in trauma has increased interest in thromboelastography (TEG®) and thromboelastometry (ROTEM®), which promptly evaluate the entire clotting process and may guide blood product therapy. Our objective was to review the evidence for their role in diagnosing early coagulopathies, guiding blood transfusion, and reducing mortality in injured patients.

**Methods:**

We considered observational studies and randomized controlled trials (MEDLINE, EMBASE, and Cochrane databases) to February 2014 that examined TEG®/ROTEM® in adult trauma patients. We extracted data on demographics, diagnosis of early coagulopathies, blood transfusion, and mortality. We assessed methodologic quality by using the Newcastle-Ottawa scale (NOS) for observational studies and QUADAS-2 tool for diagnostic accuracy studies.

**Results:**

Fifty-five studies (12,489 patients) met inclusion criteria, including 38 prospective cohort studies, 15 retrospective cohort studies, two before-after studies, and no randomized trials. Methodologic quality was moderate (mean NOS score, 6.07; standard deviation, 0.49). With QUADAS-2, only three of 47 studies (6.4%) had a low risk of bias in all domains (patient selection, index test, reference standard and flow and timing); 37 of 47 studies (78.8%) had low concerns regarding applicability. Studies investigated TEG®/ROTEM® for diagnosis of early coagulopathies (*n* = 40) or for associations with blood-product transfusion (*n* = 25) or mortality (*n* = 24). Most (*n* = 52) were single-center studies. Techniques examined included rapid TEG® (*n* =12), ROTEM® (*n* = 18), TEG® (*n* = 23), or both TEG® and rapid TEG® (*n* = 2). Many TEG®/ROTEM® measurements were associated with early coagulopathies, including some (hypercoagulability, hyperfibrinolysis, platelet dysfunction) not assessed by routine screening coagulation tests. Standard measures of diagnostic accuracy were inconsistently reported. Many abnormalities predicted the need for massive transfusion and death, but predictive performance was not consistently superior to routine tests. One observational study suggested that a ROTEM®-based transfusion algorithm reduced blood-product transfusion, but TEG®/ROTEM®-based resuscitation was not associated with lower mortality in most studies.

**Conclusions:**

Limited evidence from observational data suggest that TEG®/ROTEM® tests diagnose early trauma coagulopathy and may predict blood-product transfusion and mortality in trauma. Effects on blood-product transfusion, mortality, and other patient-important outcomes remain unproven in randomized trials.

**Electronic supplementary material:**

The online version of this article (doi:10.1186/s13054-014-0518-9) contains supplementary material, which is available to authorized users.

## Introduction

The emerging understanding of early coagulopathies and their clinical consequences after severe trauma have created a search for better coagulation assays. Current routine screening coagulation tests (RSCTs), such as activated partial thromboplastin time (aPTT) and prothrombin time (PT), have limited utility to diagnose early trauma coagulopathies and direct their treatment. Neither test predicts the extent of bleeding in critically ill or trauma patients [1], and a recent systematic review concluded that they are inappropriate for trauma [2]. The cell-based understanding of hemostasis [3], emphasizing tissue factor (TF) as the initiator of coagulation and the role of platelets, has challenged the clotting cascade concept that underlies RSCTs. The cell-based model and the need for shorter turnaround time (TAT) for tests to guide transfusion in bleeding trauma patients have propelled interest in thromboelastography (TEG®; Hemoscope Corporation, Niles, IL, USA) and thromboelastometry (ROTEM®; Tem International GmbH).

TEG® and ROTEM® are based on the principle that the result of the hemostatic process is a clot whose physical properties determine patients’ hemostatic status. These tests provide global information on the dynamics of clot development, stabilization, and dissolution, reflecting *in vivo* hemostasis, and assess both thrombosis and fibrinolysis [4]. The additional information from TEG®/ROTEM® is based on their performance in whole blood [4], whereas RSCTs are performed in plasma, without the cellular components of platelets and tissue-bearing cells.

By systematically searching for relevant studies, we sought to evaluate the evidence that the use of TEG® and ROTEM® in adult traumatically injured patients (a) diagnoses trauma coagulopathies on admission to hospital, (b) guides transfusion, and (c) reduces mortality.

## Materials and methods

This descriptive systematic review was reported in accordance with Preferred Reporting Items for Systematic Reviews and Meta-Analyses (PRISMA) guidelines [5].

### Information sources and search technique

With the assistance of an experienced librarian, we searched MEDLINE (1946 to February 2014), EMBASE (1947 to February 2014), and Cochrane Controlled Trials Register (from inception to February 2014) to identify studies of thromboelastography and thromboelastometry in trauma. We used a sensitive search strategy combining MeSH headings and the key words “thromboelastography” AND “trauma,” “thromboelastometry” AND “trauma,” “thromboelastography” AND injury,” “thromboelastometry” AND “injury,” TEG® AND “trauma,” TEG® AND “injury,” ROTEM® AND “trauma,” and ROTEM® AND “injury.” Search terms were defined *a priori* and by reviewing the MeSH terms of articles identified in preliminary literature searches. Two authors (LTL, AKS) independently reviewed the abstracts of all articles identified by the literature search and selected articles for detailed review if either reviewer considered them potentially relevant. We also searched the bibliographies of all articles selected for detailed review and all relevant published reviews to find any other studies potentially eligible for inclusion. We did not search conference proceedings. No language restrictions were imposed; we translated two studies in Spanish and Italian and engaged a medical student to translate one Chinese study that was ultimately excluded. Details of the search strategies are in Additional file 1.

### Eligibility criteria and study selection

Studies were eligible for inclusion if they were observational studies or randomized controlled trials (RCTs) that evaluated TEG®/ROTEM® in adult trauma patients and reported outcomes related to diagnosis of coagulopathies (hypocoagulation, hypercoagulation, platelet dysfunction, hyperfibrinolysis (HF), TAT), transfusion management (prediction of massive transfusion (MT), and transfusion guidance), or mortality (prediction and reduction). Studies were excluded if they enrolled only burn patients or enrolled patients in other surgical specialties, or were case reports or case series. Two independent reviewers (LTL, AKS) reviewed all full-text versions of all potentially eligible studies. Agreement between reviewers was assessed by using the Cohen κ [6]. In case of disagreement, consensus was reached by discussion with a third author (BN, NKJA).

### Data abstraction and analysis

We abstracted data from included studies on study objective, setting and study design, patient selection, clinical and demographic characteristics, TEG®/ROTEM® technique, RSCT technique, presence of comparison group, blood-product transfusion, and mortality. Two authors (LTL, AKS) independently assessed study methodology based on the Newcastle-Ottawa Scale for cohort studies [7] and QUADAS-2 [8] for quality assessment of diagnostic accuracy studies. For studies that did not report diagnostic accuracy, we supplemented the Newcastle-Ottawa scale by assessing the description of TEG®/ROTEM® performance. In applying the Newcastle-Ottawa scale, we considered management by TEG®/ROTEM® to be the relevant exposure and a nonexposed cohort to be one that was managed without TEG®/ROTEM®. We considered the following outcomes: (a) diagnostic performance of TEG®/ROTEM® parameters compared with RSCT (PT, aPTT, INR, platelet count, fibrinogen) for early coagulopathies, (b) utilization of blood products red blood cells (RBCs), fresh frozen plasma (FFP), platelets concentrate (PLT), fibrinogen concentrate (FC), cryoprecipitate, prothrombin complex concentrate (PCC), and (c) mortality. Because of clinical and methodologic heterogeneity among studies, we anticipated reporting results qualitatively instead of conducting meta-analyses.

## Results

**Figure 1:**
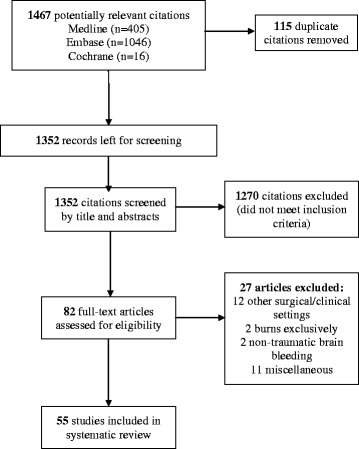
**Flow of studies through the systematic review.**

The electronic search identified 1,352 potentially relevant studies. After evaluating 82 full-text manuscripts, 55 met inclusion criteria (Figure 1). An excellent agreement was reached between the reviewers for study inclusion (κ = 0.82). References for the excluded studies are in Additional file 1.

### Study characteristics

All 55 studies were observational (Table 1). Forty studies [9-48] addressed the use of TEG®/ROTEM® in diagnosing early coagulopathies; 25 studies [9,15,16,22,23,25,26,29,31,32,35-37,41,45,47-56] examined associations with transfusion; and 24 studies [14,16,21,23,29,32,33,36-38,40,45,47,48,51,54,56-63] examined associations with mortality. Only three [40,41,61] were conducted in multiple centers. Thirty-eight studies were prospective cohorts [9-14,16,17,19,20,24-28,30,32,33,36,38,40-48,50,52,53,55-58,61,62], two were before-after [37,63], and 15 were retrospective cohorts [15,18,21-23,29,31,34,35,39,49,51,54,59,60]. The techniques used for TEG® and ROTEM® varied: in 28 studies [9,11-14,17,20,22,26,28,30,33,38-41,44,45,47,49,50,52-55,58,59,61,63], the tests were done at 37°C; in nine [10,15,16,18,19,21,37,43,57], at the patient’s temperature; in 16, there was no description of the temperature [23-25,29,31,32,34-36,42,46,48,51,56,60,62]; and one study performed the test at different temperatures [27]. Rapid TEG® (r-TEG®), a technique with fresh whole blood using a solution containing TF as the coagulation trigger, was used in 12 studies [17,18,21,25,33-35,37,43,49,52,54]; ROTEM®, in 18 studies [12,14,20,22,23,26,28,31,32,38,40,42,50,51,55,58-60]; TEG®, in 23 studies [9-11,13,15,16,19,24,27,29,30,36,39,41,44,45,53,56-58,61-63]; and two studies performed TEG® and r-TEG® [17,46] in the same cohort of patients.

**Table 1:** **Characteristics of the studies included in the systematic review**

**Reference**	**Design**	**Population/Years of enrollment**	**Patients/Centers**	**Age (years)**	**Male,** ***n*** **(%)**	**Technique (TEG®/ROTEM®)**	**Intervention**
Kaufmann 1997 [9]	Prospective	ISS 12.3^b^	69/1	40.0^b^	41 (59.4%)	TEG® - 37°C	None
		1994 - 1995				Whole blood	
						Celite activated	
Watts 1998 [10]	Prospective	ISS 16.6^b^	112/1	36.4^b^	76 (68.0%)	TEG® - patient’s T	None
		1996 - 1997				Citrated blood recalcified	
Schreiber 2005 [11]	Prospective	ISS 23.0^a^	65/1	42.0^b^	45 (69.0%)	TEG® - 37°C	None
		Years not reported				Whole blood	
						Kaolin activated	
Rugeri 2007 [12]	Prospective	ISS 22.0^a^	88/1	34.0^b^	68 (77.2%).	ROTEM® - 37°C	None
		2004				Citrate blood, recalcified	
						Ellagic acid and TF	
Nekludov 2007 [13]	Prospective	ISS TBI: 33.0^a^	47/1	TBI: 42.0^a^	19 (95.0%)	TEG® - 37°C	None
		ISS general: 46.0^a^ 2006		Trauma: 36.0^a^		Citrated blood, recalcified	
						Kaolin added	
Levrat 2008 [14]	Prospective	ISS HF: 38.0^a^	87/1	HF: 29.0^a^	HF: 64 (78.0%)	ROTEM® - 37°C	None
		ISS non HF: 20.0^a^		No HF: 30.0^a^	No HF: 4 (80.0%)	Citrated blood, recalcified	
		2004				Ellagic acid or TF	
Park 2008 [57]	Prospective	ISS 23.0^b^	58/1	47.0^b^	44 (76%)	TEG® - patient’s T	None
		2004-2005				Citrated blood, recalcified	
						Added TF	
Plotkin 2008 [15]	Retrospective	ISS 21.0^b^	44/1	Not reported	Not reported	TEG® - patient’s T	None
		2004				Fresh blood	
						Celite 1% added	
Carroll 2009 [16]	Prospective	ISS 20.0^a^	161/1	42.0^a^	118 (73.0%)	TEG® - patient’s T	None
		Years not reported				Citrated blood	
						Heparinized for PM	
Jeger 2009 [17]	Prospective	ISS 29.0^a^	20/1	48.0^a^	13 (65.0%).	r-TEG® and TEG® - 37°C	None
		Years not reported				Fresh blood	
						TF added	
Kashuk 2009 [49]	Retrospective	ISS 29.0^a^	44/1	38.9^b^	32 (69.6%)	r-TEG® - 37°C	None
		2008				Citrated and non-citrated	
						r-TEG® solution	
Kashuk 2009 [18]	Retrospective	ISS Hypercoagulable: 26^b^ ISS Normal: 24.0^b^	152/1	Hypercoagulable: 45.0^a^ Normal group: 38.0^a^	107 (70.5%)	r-TEG® - patient’s T Whole blood	None
		Years not reported				Added Kaolin and TF	
Park 2009 [19]	Prospective	ISS burn: 18.1^b^	78/1	Burn: 56.0^b^	Burn: 18 (72.0%)	TEG® - patient’s T	None
		ISS non burn: 21.7^b^ 2004 – 2005		Nonburn: 43.0^b^ Controls: 37.3^b^	Nonburn: 26 (78.0%) Control: 11 (55.0%)	Fresh blood TF added	
Schöchl 2009 [58]	Prospective	ISS 42.0^b^	33/1	45.0^a^	22 (67.0%).	ROTEM® - 37°C	None
		2003 - 2007				Citrated blood, recalcified	
						TF added	
Doran 2010 [20]	Prospective	ISS MT: 35.0^a^	25/1	21.0^a^	25 (100.0%)	ROTEM® - 37°C	None
		ISS non MT: 20.0^a^				Citrated blood, recalcified	
		2009					
Kashuk 2010 [21]	Retrospective	ISS MT: 32.5^a^	61/1	34.2^b^	Not reported	r-TEG® - patient’s T	None
		ISS ModT: 29.0^a^				Fresh whole blood	
		ISS MinT: 34.0^a^				r-TEG® solution	
		Years not reported					
Leemann 2010 [22]	Retrospective	ISS 31.1^b^	53/1	39.6^b^	40 (75.5%)	ROTEM® - 37°C	None
		2006				Citrated blood, recalcified	
						Ellagic acid or TF	
Schöchl 2010 [59]	Retrospective	ISS 38.0^b^	131/1	46.0^b^	96 (73.0%)	ROTEM® - 37°C	ROTEM® FC and PCC guided therapy
		2005 - 2009				Citrated blood, recalcified	
						Calcium chloride added	
Schöchl 2011 [23]	Retrospective	ISS survivors: 20.0^a^	88/1	47.0^a^	67 (76.0%)	ROTEM® - T not reported	None
		ISS nonsurvivors: 29.0^a^				Citrated blood, recalcified	
		2005 - 2010				TF, Kaolin, Cytochalasin	
Watters 2010 [24]	Prospective	ISS surgery: 35.3^b^	80/1	Surgery: 41.1^b^	59 (73.7%)	TEG® - T not reported	None
		ISS control: 21.2^b^		Controls: 33.7^b^		No further details reported	
		Years not reported					
Cotton 2011 [25]	Prospective	ISS 14.0^a^	272/1	34.0^a^	201 (74.0%)	r-TEG® - T not reported	None
		2009 - 2010				Citrated blood	
						CaCl^2^, Kaolin and TF added	
Davenport 2011 [26]	Prospective	ISS 12.0^a^	300/1	33.0^a^	246 (82.0%)	ROTEM® - 37°C	None
		2007 - 2009				Citrated blood, recalcified	
						TF added	
Davenport 2011 [50]	Prospective	ISS 29.0^a^	50/1	42.0^a^	41 (82.0%)	ROTEM® - 37°C	None
		2007 - 2009				Citrated blood, recalcified	
						TF added	
Differding 2011 [27]	Prospective	ISS 20.0^a^	46/1	Patients: 48.0^a^	23 (50.0%)	TEG® - 38, 36, 34, 32°C	None
		Years not reported		Controls: 38.0^a^		Citrated blood, recalcified	
						Kaolin solution added	
Jansen 2013 [28]	Prospective	ISS 19.0^a^	10/1	Not reported	Not reported	ROTEM® - 37°C	None
		2010				Citrated blood, recalcified	
						TF, Cytochalasin	
Nystrup 2011 [29]	Retrospective	ISS 21.0^b^	89/1	39.0^b^	59 (66.0%)	TEG® - T not reported	None
		2006 - 2007				Citrated blood	
						No further technical details	
Ostrowski 2011 [30]	Prospective	ISS 5–36	80/1	48.0^b^	54 (67.6%)	TEG® - 37°C	None
		2010				Citrated blood	
						No further details reported	
Schöchl 2011 [51]	Retrospective	FC-PCC group: 35.5^b^	681/2	FC-PCC: 37.3^b^	505 (74.1%)	ROTEM® - T not reported	PCC and FC guided by ROTEM®. Comparison with standard FFP transfusion
		FFP group: 35.2^b^		FFP: 39.0^b^		No technical details reported	
		2006 - 2009					
Schöchl 2011 [31]	Retrospective	MT group: 27.0^a^	323/1	44.0^a^	255 (78.9%)	ROTEM® - T not reported	None
		Non MT group: 42.0^a^				Citrated blood, recalcified	
		2005 - 2010				Kaolin, TF, Cytochalasin	
Tauber 2011 [32]	Prospective	ISS 34.0^a^	334/1	43.0^a^	260 (77.8%)	ROTEM® - T not reported	FFP, PLT, FC and PCC guided by ROTEM®.
		2005 - 2008				No further technical details	
Theusinger 2011 [60]	Retrospective	ISS trauma HF: 55^b^	35/1	55.0^b^	26 (74.2%)	ROTEM® - T not reported	None
		Non trauma HF: 43^b^				No further technical details	
		2008 - 2010					
Cotton 2012 [33]	Prospective	ISS HF: 25.0^a^	1996/1	HF group: 29.0^a^	HF: 27 (67.0%)	r-TEG® - 37°C	None
		ISS non HF: 16.0^a^		No HF: 33.0^a^	Non HF: 1466 (75.0%)	Citrated blood, recalcified	
		2009 - 2010				Kaolin and TF added	
Cotton 2012 [34]	Retrospective	ISS PE: 31.0^a^	2067/1	PE: 41.0^a^	PE group: 36 (69.0%)	r-TEG® - T not reported	None
		ISS no PE: 19.0^a^		No PE: 33.^0a^	No PE: 1530 (76%)	Citrated blood	
		2009 – 2011				Na Chloride, TF, Kaolin	
Davis 2013 [61]	Prospective	ISS 25.0^a^	50/2	48.5^a^	36 (72.0%)	TEG®. TEG®-PM - 37°C Activation solution added Heparinized blood	None
		Years not reported					
						ADP, AA and Kaolin	
Holcomb 2012 [35]	Retrospective	ISS 17.0^a^	1974/1	33.0^a^	1480 (75.0%)	r-TEG® - T not reported	None
		2009 - 2011				Citrated blood	
						CaCl^2^, kaolin and TF added	
Ives 2012 [36]	Prospective	ISS dead: 26.4^a^	118/1	Dead: 34.8^b^	91 (77.1%)	TEG®, T not reported	None
		ISS alive: 14.8^a^		Alive: 36.7^b^		Citrated blood	
		2010 - 2011				Kaolin and CaCl^2^ added	
Jeger 2012 [52]	Prospective	ISS 18.0^b^	76/1	49.0^b^	55 (72.0%)	r-TEG® - 37°C	Physicians blinded to TEG® Transfusion guided clinically and with RSCT results.
		2009 - 2010				TF, Kaolin added	
						Citrated blood, recalcified	
Kashuk 2012 [37]	Before and after study	ISS 0–25: 9%	68/1	r-TEG®: 33.3^b^	r-TEG®: 29 (85.0%)	r-TEG® - patient’s T	TEG® guided resuscitation implemented and compared with the pre TEG® period
		ISS 26–35: 29%		TEG®: 40.5^b^	TEG®: 25(74.0%)	Fresh blood	
		ISS ≥ 36: 62%				Kaolin, TF, stabilizers added	
		Over 7 months					
		Years not reported					
Kunio 2012 [62]	Prospective	ISS 21.0^a^	69/1	46.0^a^	56 (81.2%)	TEG® - T not reported	None
		2010 - 2011				Fresh whole blood non Citrated and Kaolin activated	
Kutcher 2012 [38]	Prospective	ISS 22.0^b^	115/1	40.8^b^	Not reported	ROTEM® - 37°C	None
		2011 - 2012				Citrated whole blood	
						TF and aprotinin added	
Nascimento 2012 [39]	Retrospective	ISS 26.0^b^	219/1	39.0^b^	154 (69.0%)	TEG® - 37°C	None
		2007				Citrated blood	
						Kaolin, stabilizers added	
Ostrowski 2012 [53]	Prospective	ISS heparinized: 31.0^a^	77/1	Heparin:	53 (68.8%)	TEG® - 37°C	None
		ISS non heparin: 17.0^a^		74.0^a^		Citrated whole blood	
		2010		Noneparin:		Kaolin TEG®, heparinase TEG®	
				44.0^a^			
Pezold 2012 [54]	Retrospective	ISS 29.0^b^	80/1	34.0^b^	65 (81.0%)	r- TEG® - 37°C	None
		2008 - 2010				Fresh whole blood	
						Kaolin and TF added	
Raza 2013 [55]	Prospective	ISS 10.0^a^	288/1	37.0^a^	236 (81.9%)	ROTEM® - 37°C	None
		2007 - 2009				Citrated blood, added TF Antifibrinolytic, CaCl^2^ or Aprotinin	
Rourke 2012 [40]	Prospective	ISS 34.0^a^	517/2	36.0^a^	405 (78.0%)	ROTEM® - 37°C	Pre-fixed MTP, including administration of RBC, FFP, PLT, CRYO and FC and ex vivo FC
		2008 - 2010				Citrated blood	
						Recalcified	
Wohlauer 2012 [41]	Prospective	ISS 19.0^b^	51/2	44.0^b^	32 (63.0%)	TEG® - 37°C	None
		Years nor reported				Citrated whole blood	
						Kaolin TEG®, AA and ADP TEG®-PM	
Woolley 2013 [42]	Prospective	ISS not reported	48/1	24.0^a^	Not reported	ROTEM® - T not reported	None
		2009				Citrated blood, recalcified	
						Added TF, Cytochalasin	
Chapman 2013 [43]	Prospective	Non TEG®: 18.3^a^	304/1	Non TEG®: 35.1^a^	Non TEG®: 168 (64.0%)	r-TEG® - patient’s T	None
		TEG®: 33.2^a^		TEG®: 37.7^a^	TEG®: 29 (69.0%)	Fresh whole blood	
		2009 - 2012				Kaolin and TF added	
Chapman 2013 [56]	Prospective	ISS 30.0^a^	289/1	43.0^b^	196 (68.0%)	TEG® - T not reported	None
		2010 - 2012				Citrated blood, recalcified	
						Kaolin, stabilizers added	
Harr 2013 [44]	Prospective	ISS 23.5^a^	68/1	38.0^b^	45 (66.0%)	TEG® - 37°C	None
						Citrated blood, recalcified	
						Kaolin, stabilizers added	
Johansson 2013 [45]	Prospective	ISS 17.0^b^	182/1	43.0^b^	136 (75.0%)	TEG® - 37°C	MTP (1:1:1 ratio) initially and guided by TEG® thereafter. Also TXA, CRYO and FC administered.
		2010 - 2011				Citrated blood, recalcified	
						Kaolin and TF activated and Functional fibrinogen test	
Lee 2013 [46]	Prospective	ISS 17.0^b^ 2010 - 2012	190/1	43.0^b^	136 (71.6%)	TEG®, r-TEG® - T not reported	None
						Citrated blood, recalcified	
						Kaolin and TF activated	
Tapia 2013 [63]	Before and after study	ISS 25.0^b^	289/1	35.0^b^	251 (86.8%)	TEG® - 37°C	TEG® guided resuscitation pre MTP and guided by MTP without TEG® thereafter
		2008 - 2010				Whole blood	
						Celite activated	
Kornblith 2014 [47]	Prospective	ISS 9.0^a^	251/1	35.0^a^	202 (80.7%)	TEG® - 37°C	None
		Years not reported				Citrated blood, recalcified	
						Kaolin and TF activated and functional fibrinogen test	
Branco 2014 [48]	Prospective	ISS 1 - 51	118/1	36.9^b^	97 (77.1%)	TEG® - T not reported	None
		2011				Citrated blood, recalcified	
						Kaolin added	

^a^Median, AA – arachidonic acid, ADP – adenosine diphosphate, AIS – abbreviated injury score, ^b^Mean, CaCl^2^ – calcium chloride, CRYO – cryoprecipitate, DVT – deep vein thrombosis, FC – fibrinogen concentrate, FFP – fresh frozen plasma, GCS – Glasgow Coma Scale, HCR – hemostatic control resuscitation, HF – hyperfibrinolysis, ISS – injury severity score, LMWH – low molecular weight heparin, MinT – minimal transfusion, ModT – moderate transfusion, MT – massive transfusion, MTP – massive transfusion protocol, Na – sodium, PCC – prothrombin complex concentrate, PE – pulmonary embolism, PLT – platelets, PM – platelet mapping, RBC – red blood cells, ROTEM® - rotational thromboelastometry, RSCT – routine screening coagulation tests, r-TEG® - rapid thromboelastography, TBI – traumatic brain injury, TEG® - thromboelastography, TF – tissue factor, TXA – tranexamic acid.

### Demographic data

The 55 studies included 12,489 patients. The mean or median age of the patients ranged from 24 to 74 years (mean of mean or median age across all studies, 40 years). The majority were male (9,858 patients, 78.9%), and the mean injury severity score (ISS) [64] ranged from 9 to 55 (mean of means in all studies, 25.3). Studies included trauma with or without traumatic brain injury (TBI) (*n* = 49), isolated TBI (*n* = 4), and burns with all types of trauma (*n* = 2). The median (interquartile range (IQR)) sample size of the included studies was 87 (52 to 235).

### Methodologic quality

The overall methodologic quality of the studies was moderate (Table 2). Most studies (*n* = 36) included consecutive patients [10,12-14,16,19,21-27,30-35,37-41,45,46,50-52,54-56,58-60,63]. Fifty-two (94.5%) studies were not controlled [9-11,13-36,38-50,52-62]. The three (5.5%) controlled studies [37,51,63] had comparable control groups managed without TEG®/ROTEM®. Eleven (20%) studies used healthy [12,13,19,27,41,42,44,61] or other hospitalized trauma [24,32,60] controls to examine associations between TEG®/ROTEM® abnormalities and outcomes. Nearly all (*n* = 53, 96.4%) studies had adequate follow-up. The mean Newcastle-Ottawa score (*n* = 55 studies) was 6.07 (SD, 0.49; possible range, 1 to 9).

**Table 2:** **Newcastle-Ottawa score** [6] **for the cohort studies included in the systematic review**

**Reference**	**Representativeness of the exposed cohort**	**Selection of non-exposed cohort**	**Ascertainment of exposure**	**Outcome not present at start**	**Comparability of controls**	**Assessment of outcome**	**Adequate follow-up duration**	**Loss to follow-up**	**Total score**
Kaufmann 1997 [9]	*	-	*	*	-	*	*	*	6/9
Watts 1998 [10]	*	-	*	*	-	*	*	*	6/9
Schreiber 2005 [11]	*	-	*	*	-	*	*	*	6/9
Rugeri 2007 [12]	*	-	*	*	-	*	*	*	6/9
Nekdulov 2007 [13]	*	-	*	*	-	*	*	*	6/9
Levrat 2008 [14]	*	-	*	*	-	*	*	*	6/9
Park 2008 [57]	*	-	*	*	-	*	*	*	6/9
Plotkin 2008 [15]	*	-	*	*	-	*	*	*	6/9
Carroll 2009 [16]	*	-	*	*	-	*	*	*	6/9
Jeger 2009 [17]	*	-	*	*	-	*	*	*	6/9
Kashuk 2009 [49]	*	-	*	*	-	*	*	*	6/9
Kashuk 2009 [18]	*	-	*	*	-	*	*	*	6/9
Park 2009 [19]	*	-	*	*	-	*	*	*	6/9
Schöchl 2009 [58]	*	-	*	*	-	*	*	*	6/9
Doran 2010 [20]	*	-	*	*	-	*	*	-	5/9
Kashuk 2010 [21]	*	-	*	*	-	*	*	*	6/9
Leemann 2010 [22]	*	-	*	*	-	*	*	*	6/9
Schochl 2010 [59]	*	-	*	*	-	*	*	*	6/9
Schochl 2011 [23]	*	-	*	*	-	*	*	*	6/9
Watters 2010 [24]	*	-	*	*	-	*	*	*	6/9
Cotton 2011 [25]	*	-	*	*	-	*	*	*	6/9
Davenport 2011 [26]	*	-	*	*	-	*	*	*	6/9
Davenport 2011 [50]	*	-	*	*	-	*	*	-	5/9
Differding 2011 [27]	*	-	*	*	-	*	*	*	6/9
Jansen 2011 [28]	*	-	*	*	-	*	*	*	6/9
Nystrup 2011 [29]	*	-	*	*	-	*	*	*	6/9
Ostrowski 2011 [30]	*	-	*	*	-	*	*	*	6/9
Schöchl 2011 [51]	*	*	*	*	*	*	*	*	8/9
Schöchl 2011 [31]	*	-	*	*	-	*	*	*	6/9
Tauber 2011 [32]	*	-	*	*	-	*	*	*	6/9
Theusinger 2011 [60]	*	-	*	*	-	*	*	*	6/9
Cotton 2012 [33]	*	-	*	*	-	*	*	*	6/9
Cotton 2012 [34]	*	-	*	*	-	*	*	*	6/9
Davis 2013 [61]	*	-	*	*	-	*	*	*	6/9
Holcomb 2012 [35]	*	-	*	*	-	*	*	*	6/9
Ives 2012 [36]	*	-	*	*	-	*	*	*	6/9
Jeger 2012 [52]	*	-	*	*	-	*	*	*	6/9
Kashuk 2012 [37]	*	*	*	*	*	*	*	*	8/9
Kunio 2012 [62]	*	-	*	*	-	*	*	*	6/9
Kutcher 2012 [38]	*	-	*	*	-	*	*	*	6/9
Nascimento 2012 [39]	*	-	*	*	-	*	*	*	6/9
Ostrowski 2012 [53]	*	-	*	*	-	*	*	*	6/9
Pezold 2012 [54]	*	-	*	*	-	*	*	*	6/9
Raza 2013 [55]	*	-	*	*	-	*	*	*	6/9
Rourke 2012 [40]	*	-	*	*	-	*	*	*	6/9
Wohlauer 2012 [41]	*	-	*	*	-	*	*	*	6/9
Woolley 2013 [42]	*	-	*	*	-	*	*	*	6/9
Chapman 2013 [43]	*	-	*	*	-	*	*	*	6/9
Chapman 2013 [56]	*	-	*	*	-	*	*	*	6/9
Harr 2013 [44]	*	-	*	*	-	*	*	*	6/9
Johansson 2013 [45]	*	-	*	*	-	*	*	*	6/9
Lee 2013 [46]	*	-	*	*	-	*	*	*	6/9
Tapia 2013 [63]	*	*	*	*	*	*	*	*	8/9
Kornblith 2014 [47]	*	-	*	*	-	*	*	*	6/9
Branco 2014 [48]	*	-	*	*	-	*	*	*	6/9

Refer to reference [7] for a description of Newcastle-Ottawa Quality Assessment Scale for cohort studies. In general, more stars denote higher quality. “Representativeness” is awarded a star if the cohort is truly or somewhat representative of the population of interest. For selection of the nonexposed cohort, a star is awarded if it is drawn from the same population as the exposed cohort. The relevant exposure in this review is management by using TEG®/ROTEM®; we considered a non-exposed cohort to be one that was managed without TEG®/ROTEM®; several other studies [12,13,19,24,27,32,41,42,44,60,61] used healthy or other hospitalized controls to examine associations between TEG®/ROTEM® abnormalities and outcomes. Exposure is satisfactorily ascertained if data are collected from a secure record. A star is awarded if the outcome is not present at the start of the study. A maximum of two stars can be given for “Comparability of controls” for controlling of confounders in either the design (matching) or analysis (statistical adjustment) phase. We also gave one star when selection criteria appeared to create comparable groups via restriction. “Assessment of outcome” is awarded a star if the outcomes were assessed by independent blind assessment or record linkage; we also considered the outcome of mortality to be adequately assessed in all studies reporting it was due to low risk of bias. The duration of follow-up was considered adequate if it was long enough for the outcomes to occur. Completeness of follow-up was considered adequate if all patients were accounted for or if the number lost to follow-up was sufficiently low to be unlikely to introduce bias.

We assessed 47 studies of diagnostic accuracy by using QUADAS-2 (Table 3). Considering the domains of patient selection, index test, reference standard, and flow and timing, only three studies (6.4%) [32,37,39] had low risk of bias in all; 24 (51.1%) [10,16,21-26,30,31,33-35,38,40,44,45,47,50,52,54,55,58,62] had low and unclear risks; and 20 (42.5%) [9,11-15,17-20,29,36,42,48,49,53,57,59-61] had high risk of bias in at least one domain. In the applicability section, 37 studies (78.8%) [9-11,16-18,21-26,29-40,44,45,47,48,50,52-55,57-59,62] had low concerns, and 10 studies (21.2%) [12-15,19,20,42,49,60,61] had at least one domain with high concern. The remaining eight studies that that did not evaluate diagnostic performance against a reference standard are represented in Table 4.

**Table 3:** **Assessment of studies of diagnostic performance of TEG®/ROTEM® by using the QUADAS-2** [8] **tool**

	**Risk of bias**				**Applicability concerns**		
**Reference**	**Patient selection**	**Index test**	**Reference standard**	**Flow and timing**	**Patient selection**	**Index test**	**Reference standard**
Kaufmann 1997 [9]	?	↑	↑	↓	↓	↓	↓
Watts 1998 [10]	↓	?	↓	↓	↓	↓	↓
Schreiber 2005 [11]	↑	?	?	↓	↓	↓	↓
Rugeri 2007 [12]	↑	↓	↓	↓	↑	↓	↓
Nekludov 2007 [13]	↑	?	?	↓	↑	↓	↓
Levrat 2008 [14]	↓	?	↑	↓	↓	↓	↑
Park 2008 [57]	↑	?	?	↓	↓	↓	↓
Plotkin 2008 [15]	↑	?	?	↑	↑	↓	↓
Carroll 2009 [16]	↓	?	?	↓	↓	↓	↓
Jeger 2009 [17]	↑	?	?	↓	↓	↓	↓
Kashuk 2009 [49]	↑	?	↑	↓	↑	↓	↓
Kashuk 2009 [18]	↑	?	?	↓	↓	↓	↓
Park 2009 [19]	↑	?	?	↓	↑	↓	↓
Schöchl 2009 [58]	↓	?	?	↓	↓	↓	↓
Doran 2010 [20]	↑	?	?	↓	↑	↓	↓
Kashuk 2010 [21]	↓	?	?	↓	↓	↓	↓
Leemann 2010 [22]	↓	?	?	↓	↓	↓	↓
Schochl 2010 [59]	↑	↓	↓	↓	↓	↓	↓
Schochl 2011 [23]	↓	?	?	↓	↓	↓	↓
Watters 2010 [24]	↓	?	?	↓	↓	↓	↓
Cotton 2011 [25]	↓	?	?	↓	↓	↓	↓
Davenport 2011 [26]	↓	?	?	↓	↓	↓	↓
Davenport 2011 [50]	↓	?	?	↓	↓	↓	↓
Nystrup 2011 [29]	↑	?	?	↓	↓	↓	↓
Ostrowski 2011 [30]	↓	?	?	↓	↓	↓	↓
Schöchl 2011 [31]	↓	?	?	↓	↓	↓	↓
Tauber 2011 [32]	↓	↓	↓	↓	↓	↓	↓
Theusinger 2011 [60]	↑	?	?	↓	↑	↓	↓
Cotton 2012 [33]	↓	?	?	↓	↓	↓	↓
Cotton 2012 [34]	↓	?	?	↓	↓	↓	↓
Davis 2013 [61]	↑	?	?	↓	↑	↓	↓
Holcomb 2012 [35]	↓	?	?	↓	↓	↓	↓
Ives 2012 [36]	↑	?	?	↓	↓	↓	↓
Jeger 2012 [52]	↓	?	↓	↓	↓	↓	↓
Kashuk 2012 [37]	↓	↓	↓	↓	↓	↓	↓
Kunio 2012 [62]	?	?	?	↓	↓	↓	↓
Kutcher 2012 [38]	↓	?	?	↓	↓	↓	↓
Nascimento 2012 [39]	↓	↓	↓	↓	↓	↓	↓
Ostrowski 2012 [53]	↑	?	?	↓	↓	↓	↓
Pezold 2012 [54]	↓	?	?	↓	↓	↓	↓
Raza 2013 [55]	↓	?	?	↓	↓	↓	↓
Rourke 2012 [40]	↓	?	?	↓	↓	↓	↓
Woolley 2013 [42]	↑	?	?	↓	↑	↓	↓
Harr 2013 [44]	?	?	?	↓	↓	↓	↓
Johansson 2013 [45]	↓	?	?	↓	↓	↓	↓
Kornblith 2014 [47]	?	?	?	↓	↓	↓	↓
Branco 2014 [48]	↑	?	?	↓	↓	↓	↓

We assessed studies using QUADAS-2 [8] if they evaluated diagnostic performance of TEG®/ROTEM® compared with standard laboratory tests. ↑ denotes high risk of bias, ↓ denotes low risk of bias, and ? denotes unclear risk of bias.

**Table 4:** **Details of TEG®/ROTEM® for studies that did not evaluate diagnostic performance against a reference standard**

**Reference**	**Performed in a timely manner**	**All results reported**	**Technique described in detail**	**Independently interpreted from routine screening coagulation tests**
Differding 2011 [27]	Yes	Yes	Yes	Yes
Jansen 2013 [28]	Yes	Yes	No	Yes
Schöchl 2011 [51]	Yes	Yes	No	Yes
Wohlauer 2012 [41]	Yes	Yes	Yes	Yes
Chapman 2013 [43]	Yes	Yes	Yes	Yes
Chapman 2013 [56]	Yes	No	No	Yes
Lee 2013 [46]	Yes	Yes	No	Yes
Tapia 2013 [63]	Yes	Yes	Yes	Yes

### Outcomes

The included studies collectively reported on hypocoagulability, hypercoagulability, platelet dysfunction, and hyperfibrinolysis. We summarize the parameters of TEG®/ROTEM® used for diagnosis, turnaround times (TATs), and results concerning prediction, reduction, or guidance of transfusion and prediction or reduction of mortality. Details of all studies without a control group (for clinical outcomes) or a reference standard (for diagnostic performance) are given in Table 5.

**Table 5:** **Main findings of the included studies**

**Reference**	**1. Findings on diagnosis**	**2. Findings on transfusion**	**3. Findings on mortality**
Kaufmann 1997 [9]	1. Of 69 patients, 45 were hypercoagulable (mean ISS 13.1) and seven were hypocoagulable (mean ISS, 28.6) by TEG®. Only one was hypocoagulable by elevated PT/aPTT, and two were hypercoagulable by elevated PLT		
	2. Only ISS (*P* < 0.001) and TEG® (*P* < 0.05) predicted transfusion within the first 24 h after injury. Six of the seven hypocoagulable patients received blood within the first 24 hours		
	3. None		
Watts 1998 [10]	1. Hypothermic patients (34°C) presented significantly lower TEG® α-angle, K and MA values (*P* < 0.001) even though platelet count, PT, and aPTT were within normal range, and correlated with fluid and blood transfusion.		
	2. None		
	3. None		
Schreiber 2005 [11]	1. INR and aPTT failed to detect early hypercoagulability, showing that TEG® is more sensitive. Women are more hypercoagulable than men within the first 24 hours.		
	2. None		
	3. None		
Rugeri 2007 [12]	1. Significant correlation between PT - A15-EXTEM, between aPTT - CFT-INTEM, between fibrinogen - A10-FIBTEM and between PLT - A15-INTEM. A cut off value of A15-EXTEM at 32 mm and A10-FIBTEM at 5 mm presented a good sensitivity (87 and 91%) and specificity (100 and 85%) to detect PT >1.5 and a fibrinogen less than 1 g/L, respectively.		
	2. None		
	3. None		
Nekdulov 2007 [13]	1. TBI patients had a lower PLT count (180 ± 68 × 109; mean ± SD) and longer bleeding time (674 ± 230 sec) than healthy controls (256 ± 43 × 109, *p* < .01) and (320 ± 95 sec, *p* < .005) respectively. TEG®-PM showed reduced PLT response to AA and ADP (0-86%, mean 22%) compared to healthy controls (57-89%, mean 73%).		
	2. None		
	3. None		
Levrat 2008 [14]	1. MCF showed the best correlation with the ELT when compared with amplitude and CLI. HF patients also had greater ROTEM® abnormalities, lower INR, lower fibrinogen levels and were more severely injured (↑ ISS) than the control group (all *p* < .05).		
	2. None		
	3. Patients with hyperfibrinolysis had higher mortality rate (100%, CI: 48-100% vs. 11% CI: 5-20%)		
Park 2008 [57]	1. None		
	2. None		
	3. Multiple logistic regression analysis identified MA as an independent risk factor for death, AUC ROC 0.961 (95% CI, 0.891, 1.000)		
Plotkin 2008 [15]	1. Increased K time, reduced α-angle and decreased MA demonstrated hypocoagulation.		
	2. INR, PT and aPTT did not correlate with the use of blood products (*r* = .57, *p* < .01). MA correlated with blood product use as well as PLT count. Patients with reduced MA used more blood products and had reduced PLT counts and hematocrit.		
	3. None		
Carroll 2009 [16]	1. TEG® parameters did not change significantly from the ED sampled to OR samples.		
	2. Abnormal MA-ADP at 30 min correlated with the need for transfusion (*p* = .004).		
	3. R and MA correlated importantly with fatality (both p < .001). HF was an independent predictor of fatality (*p* = .001 by chi square testing).		
Jeger 2009 [17]	1. Strong correlations between the values of K, alpha angle and MA (*p* < 0.01). Moderate correlation between K and both INR and PLT count and between MA and both INR and PLT count (*p* < 0.05). There was decrease in the time for TEG® results with r-TEG®.		
	2. None		
	3. None		
Kashuk 2009 [49]	1. None		
	2. Lab tests triggers result in blood product administration in 73.1% compared with 53.9% based on r-TEG® thresholds (*p* = .03). FFP administration guided by INR triggers would have been higher (61.5% by INR triggers versus 26.9% by r-TEG®-ACT triggers, *p* = .003).		
	3. None		
Kashuk 2009 [18]	1. 67% of patients were hypercoagulable by r-TEG®. 19% of the hypercoagulable group suffered a TE, and 12% had TE predicted by prior r-TEG®. No patients with normal coagulability by r-TEG® had an event (*p* < .001). G value was the strongest predictor of TE after controlling for thromboprophylaxis (OR: 1.25, 95% CI: 1.12-1.39). For every 1 dyne/cm2 increase in G, the *odds ratio* of a TE increased by 25%.		
	2. None		
	3. None		
Park 2009 [19]	1. PT and aPTT were prolonged compared with controls (*p* < .05). All other parameters showed hypercoagulability (low protein C, high fibrinogen level and low TAT levels). MA and α-angle were also higher compared with controls (*p* < .05). PT and aPTT in this population were increased and did not detect hypercoagulability, which was demonstrated by TEG®.		
	2. None		
	3. None.		
Schöchl 2009 [58]	1. None		
	2. None.		
	3. Prolonged CFT and lower PLT contribution to MCF were associated with increased mortality (*p* = .042 and *p* = .026 respectively). The observed mortality was higher than the expected mortality as per TRISS (88 vs. 70%, *p* = .039).		
Doran 2010 [20]	1. MCF was abnormal in all MTP cases. A10 is subsequently associated with an abnormal MCF. 64% of all patients were coagulopathic by TEM trace and only 10% had abnormal lab tests (*p* = .0005).		
	2. None		
	3. None		
Kashuk 2010 [21]	1. 34% of injured patients requiring MT had PF (ANOVA, *p* < .0001). PF occurred early (median 58 minutes). Every 1 unit drop in G increased the risk of PF by 30%		
	2. None		
	3. The risk of death correlated significantly with PF (*p =* .026) and every 1 unit drop in G increased risk of death by 10%.		
Leemann 2010 [22]	1. MT patients had significantly altered ROTEM® values on admission compared with non-MT patients. An increase in the CFT (*p =* .001), a shortening of the MCF (*p <* .001), and a shortening of the amplitude at all time-points (10/20/30 minutes) were observed in MT patients.		
	2. Variables independently associated with MT included a hemoglobin level <10 g/dL and an abnormal MCF value (AUC ROC 0.831 [95% CI: 0.719–0.942).		
	3. None		
Schochl 2010 [59]	1. None		
	2. None		
	3. The difference in mortality, after excluded patients with TBI, was 14% observed versus 27.8% predicted by TRISS and 24.3% predicted by RISC. The study shows a favorable survival rate.		
Schochl 2011 [23]	1. ROTEM® analysis revealed shorter clotting times in EXTEM and INTEM (*p* < .001), shorter CFT in EXTEM and INTEM (*p* < .0001), and higher MCF in EXTEM, INTEM, and FIBTEM (*p* < .01) in survivors compared with non-survivors, in severe isolated TBI.		
	2. According to the degree of coagulopathy, non-survivors received more RBC (*p* = .016), fibrinogen concentrate (*p* = .01), and PCC (*p* < .001) within 24 h of arrival in the ED.		
	3. Logistic regression analysis revealed EXTEM with cytochalasin D (FIBTEM) MCF and aPTT to have the best predictive value for mortality.		
Watters 2010 [24]	1. Cloth strength baseline and at follow up were elevated in the splenectomy group and not in the control group (*p* < .01). Platelet count, fibrinogen, aPTT were also elevated in the splenectomy group. In this population TEG® and RSCT were able to diagnose hypercoagulability together.		
	2. None		
	3. None		
Cotton 2011 [25]	1. Early r-TEG® values (ACT, *k*-time, and *r*-value) were available within 5 min. Late r-TEG® values (MA and α-angle) within 15 min, and RSCTs within 48 min (*p* < .001). ACT, *r*-value, and *k*-time showed strong correlation with PT, INR and aPTT whereas and α-angle correlated with platelet count (both *p* < .001).		
	2. Linear regression demonstrated that ACT predicted RBC, plasma and PLT transfusions within the first 2 h of arrival. Controlling for all demographics and ED vitals, ACT > 128 predicted MT in the first 6 h. In addition, ACT < 105 predicted patients who did not receive any transfusions in the first 24 h.		
	3. None		
Davenport 2011 [26]	1. CFT, α, A5 and MCF are significantly different in the group with coagulopathy. A5 ≤ 35 mm detects great percentage of patients with coagulopathy with lower false positive rates than PT (detected 77% of ATC, with 13% false positive).		
	2. Patients with A5 ≤ 35 mm were more likely to receive RBC (46% vs. 17%, *p* < .001) and FFP (37% vs. 11%, *p* < .001) transfusions. A5 identified patients who would require MT (rate of 71% vs. 43% for INR > 1.2, p < .001).		
	3. None		
Davenport 2011 [50]	1. None		
	2. Coagulation profile deteriorated with low FFP:PRBC ratios <1:2. Maximal hemostatic effect was observed in the 1:2 to 3:4 groups: 12% decrease in PT (*p* = .006), 56% decrease CT (*p* = .047), and 38% increase in MCF (*p* = .024). Transfusion with ≥1:1 ratio did not confer any additional improvement. There was a marked variability in response to FFP, and hemostatic function deteriorated in some patients exposed to 1:1 ratios. The beneficial effects of plasma were confined to patients with coagulopathy.		
	3. None		
Differding 2011 [27]	1. R increased (*p* < .001) and α-angle decreased (*p* < .01) in both groups (patients and controls) as T°C decreased. Between groups, R, α-angle, and MA were significantly different at each T°C (*p <* .01), with patients being more hypercoagulable. R and α-angle were more affected by T°C in controls compared with patients (*p <* .02). Temperature did not alter coagulability in the range studied in trauma patients while in the controls it did change.		
	2. None		
	3. None		
Jansen 2013 [28]	1. Repeated ROTEM® tests on samples stored at 37°C for a median of 51 minutes, show improved MCF (22 mm vs. 54 mm, *p* < .001) and α-angle (30.5 vs. 59.5°, *p* = .004) when compared to analysis at the moment of venipuncture.		
	2. None		
	3. None.		
Nystrup 2011 [29]	1. Patients with a reduced MA (<50 mm) evaluated by TEG®, presented with a higher ISS - 27 (95% CI, 20–34) vs. 19 (95% CI, 17–22), than the rest of the cohort.		
	2. MA correlated with the amount of RBC (*p* = .01), FFP (*p* = .04) and PLT (*p* = .03) transfused during the first 24 h of admission.		
	3. Patients with ↓ MA demonstrated ↑ 30-day mortality (47% vs. 10%, *p* < .001). By logistic regression ↓MA was an independent predictor of ↑ mortality after adjusting for age and ISS.		
Ostrowski 2011 [30]	1. Fibrinogen and PLT count were associated independently with clot strength in patients with ISS ≤ 26 whereas only fibrinogen was associated independently with clot strength in patients with ISS > 26. In patients with ISS > 26, adrenaline and sCD40L were independently negatively associated with clot strength.		
	2. None		
	3. None		
Schöchl 2011 [51]	1. None		
	2. RBC transfusion was avoided in 29% of patients in the fibrinogen-PCC group compared with only 3% in the FFP group (*p* < .001). Transfusion of PLT was avoided in 91% of patients in the fibrinogen-PCC group, compared with 56% in the FFP group (*p* < .001).		
	3. Mortality was comparable between groups: 7.5% in the fibrinogen-PCC group and 10.0% in the FFP group (*p* = .69).		
Schöchl 2011 [31]	1. EXTEM and INTEM CT and CFT were significantly prolonged and MCF was significantly lower in the MT group versus the non-MT group (*p* < .0001 for all comparisons).		
	2. Of patients admitted with FIBTEM MCF 0 to 3 mm, 85% received MT. The best predictive values for MT were provided by hemoglobin and Quick value (AUC ROC: 0.87 for both parameters). Similarly high predictive values were observed for FIBTEM MCF (0.84) and FIBTEM A10 0.83).		
	3. None		
Tauber 2011 [32]	1. In patients with or without TBI, the prevalence of low fibrinogen, impaired fibrin polymerization and reduced MCF was 26%, 30%, and 22%, respectively, and thus higher than the prolonged INR (14%). All patients showed ↑ F1 + 2 and TAT and low AT levels, indicating ↑ thrombin formation.		
	2. MCF FIBTEM correlated with RBC transfusion (OR 0.92, 95% CI 0.87–0.98).		
	3. ROTEM® parameters correlated with RSCTs and with mortality (FIBTEM and EXTEM MCF (*p =* .006 and *p = .*001 respectively). EXTEM MCF was independently associated with early mortality. HF ↑ fatality rates and occurred as frequently in isolated TBI as in polytrauma.		
Theusinger 2011 [60]	1. None		
	2. None		
	3. Mortality in the trauma HF group (77% ± 12%) as diagnosed by ROTEM® was significantly higher than in the nontrauma HF group (41% ± 10%, 95% CI 5%–67%) and the matched trauma group (33% ± 10%, 95% CI 13%–74%). HF is significantly (*p* = .017) associated with mortality in trauma patients.		
Cotton 2012 [33]	1. Controlling for ISS and BD on arrival, pre-hospital fluid was associated with a significant ↑in likelihood of HF. Each additional liter of crystalloid was associated with a 15% ↑ OR of HF. The *in vitro* model found that hemodilution to 15% of baseline and TF + t-PA was required to achieve an LY30 of 50%.		
	2. None		
	3. Compared with patients without HF, the HF group had higher mortality (76% vs. 10%); all *p* < .001.		
Cotton 2012 [34]	1. The PE group had admission higher MA (66 vs. 63, *p* = .05) and higher ISS (median, 31 vs. 19, *p* = .002). When controlling for gender, race, age, and ISS, elevated MA at admission was an independent predictor of PE with an OR of 3.5 for MA > 65 and 5.8 for MA > 72.		
	2. None		
	3. None		
Davis 2013 [61]	1. None		
	2. None		
	3. Median ADP inhibition of platelet function, as measured by TEG® platelet-mapping analysis, was significantly greater in TBI non-survivors (91.7%) compared to survivors (48.2%) (*p* = .035).		
Holcomb 2012 [35]	1. Overall, r-TEG® correlated with RSCTs, and could replace RSCTs on admission.		
	2. ACT-predicted RBC transfusion, and the *α*-angle predicted massive RBC transfusion better than PT, aPTT or INR (*p <* .001). The *α*-angle was superior to fibrinogen for predicting FFP transfusion (*p <* .001); MA was superior to PLT count for predicting PLT transfusion (*p <* .001); and LY-30 documented fibrinolysis. These correlations improved for transfused, shocked or TBI patients.		
	3. None		
Ives 2012 [36]	1. By the 6-h sampling, 8 (61.5%) of the HF patients (detected by TEG® parameters) had died from hemorrhage. Survivors at this point demonstrated correction of coagulopathy.		
	2. Compared with patients without HF, patients with HF had a greater need for MT (76.9% vs. 8.7%; adjusted OR = 19.1; 95% CI, 3.6 - 101.3)		
	3. On LR, HF was a strong predictor of early mortality (OR = 25.0; 95% CI, 2.8- 221.4), predicting 53% of early deaths. Patients with HF had ↑ early mortality (69.2% vs. 1.9%; adjusted OR = 55.8; 95% CI, 7.2-432.3) and in-hospital mortality (92.3% vs. 9.5%; adjusted OR = 55.5; 95% CI, 4.8 - 649.7).		
Jeger 2012 [52]	1. None		
	2. RSCTs correlate moderately with r-TEG® parameters (*R*: 0.44–0.61). Kaolin and r-TEG® were more sensitive than RSCTs and the r-TEG® *α*-angle was the parameter with the greatest sensitivity (84%) and validity (77%) at a cut-off of 74.7 degrees. When the r-TEG® *α*-angle was combined with HR *>*75 bpm, or hematocrit *<* 41%, sensitivity (84%, 88%) and specificity (75%, 73%) were improved. Cut-off points for transfusion can be determined with r-TEG® α angle and can provide better sensitivity than RSCTs.		
	3. None		
Kashuk 2012 [37]	1. INR at 6 h did not discriminate between survivors and non survivors (*p* = .10).		
	2. In r = TEG®-guided transfusion, patients with a MRTG > 9.2 received significantly less components of RBCs, FFP, and Cryo (*p* = .048, *p* = .03, and *p* = .04, respectively		
	3. r-TEG® G value was associated with survival as was MRTG and TG (*p* = .03).		
Kunio 2012 [62]	1. None		
	2. None		
	3. In TBI patients, prolonged R time (>9 min) or reduced MA (<55 mm) as evaluated by TEG®, are associated with greater mortality (50% vs. 11.7% and 33.3% vs. 9.8%, respectively; *p* = .04).		
Kutcher 2012 [38]	1. Patients with HF diagnosed by ROTEM® had lower T°C, pH, PLT count and higher INR, aPTT and D-dimer. The presence of hypothermia (temperature < 36.0°C), acidosis (pH < 7.2), relative coagulopathy (INR > 1.3 or aPTT > 30), or relative low PLT count (<200) identified HF by ROTEM® with 100% sensitivity and 55.4% specificity (AUC, 0.77).		
	2. None		
	3. HF as detected by ROTEM® was associated with MODS (63.2% vs. 24.6%, *p* = .004) and mortality (52.2% vs. 12.9%, *p* < .001).		
Nascimento 2012 [39]	1. For detection of coagulopathy, overall, TEG®-R performed worse than INR. TEG®-R had a sensitivity of 33% (95% CI, 16%-55%), specificity of 95% (95% CI, 91%-98%), PPV of 47% (95% CI, 23%-72%), and NPV of 92% (95% CI, 87%-95%). An INR of 1.5 or greater had a sensitivity of 67% (95% CI, 45%-84%), specificity of 98% (95% CI, 96%-99.7%), PPV of 84% (95% CI, 60%-97%), and NPV of 96% (95% CI, 92%-98%). An INR of 1.3 or greater also had better sensitivity, PPV, and NPV, than TEG®.		
	2. None		
	3. None		
Ostrowski 2012 [53]	1. None		
	2. Patients considered coagulopathic (“endogenous heparinization”) based on TEG® parameters (R, K, *α-*angle and MA) received more RBC (10 vs. 0), FFP (7 vs. 0) and platelet (3 vs. 0) in the first 24 hours (*p* < .05).		
	3. These patients showed a tendency towards higher 30-day mortality (50% vs. 16%, *p* = .15).		
Pezold 2012 [54]	1. None		
	2. INR, ISS, and G were predictors of MT. The predictive power for outcome MT did not differ among INR (adjusted AUC ROC = .92), aPTT (AUC ROC = .90, *p* = .41), or G (AUC ROC = .89, *p* = .39).		
	3. 21% of patients died of MT-related complications. Age, ISS, SBP, and G were associated with MT-death. For outcome MT-death, G had the greatest adjusted AUC ROC (0.93) compared with the AUC ROC for BD (0.87, *p* = .05), INR (0.88, *p* = .11), and PTT (0.89; *p* = .19).		
Raza 2013 [55]	1. None		
	2. Patients with moderate and severe fibrinolytic activity, based on plasmin-antiplasmin complex levels and ROTEM® ML > 15%, required more transfusions: RBC (2.0 and 6.5 units, respectively), FFP (1 and 2.9 units, respectively), platelets (0.2 and 0.7 units, respectively) and cryoprecipitate (0.2 and 0.6 units, respectively) (*p* < .05 for all comparisons).		
	3. Similarly, patients with moderate and severe fibrinolytic activity, had significantly greater 28-day mortality (12.1% and 40% respectively, *p* < .05).		
Rourke 2012 [40]	1. ROTEM® parameters correlated with fibrinogen level, and ex vivo fibrinogen administration reversed coagulopathy by ROTEM®		
	2. None		
	3. Fibrinogen level was an independent predictor of mortality at 24 h and 28 days (*p* < .001). Hypofibrinogenemia can be detected early by ROTEM® and administration of cryo or fibrinogen concentrate can improve survival.		
Wohlauer 2012 [41]	1. In trauma patients, median ADP inhibition of platelet function was 86.1% vs. 4.2% and impaired platelet function in response to AA was 44.9% vs. 0.5% when compared to healthy volunteers (*p* < .0001).		
	2. ADP inhibition correlated with the RBC transfusion within the first 6 hours, 59.6% (0 RBC) vs. 96.1% (>1 RBC) (Wilcoxon *p* = .025).		
	3. None.		
Woolley 2013 [42]	1. 51% of all 48 patients were coagulopathic. EXTEM MCF < 40 mm and interim EXTEM A5 and A10 predicted coagulopathy with sensitivities/specificities of 96%/58% (A5) and 100%/ 70% (A10). In addition, statistical comparison of clotting domains between normal volunteers and trauma patients suggests a difference in clot strengths due to a difference in PLT function rather than PLT number (mean 142,000/mm^3^).		
	2. None		
	3. None		
Chapman 2013 [43]	1. Both G and MA values initially normal, crossed to the hypercoagulable range at 48 hours. G values rose from 7.4 ± 0.5 Kd/cs to 15.1 ± 1.9 Kd/cs (*p* < .01), and MA from 57.6 mm to 74.5 mm (*p* = .01).		
	2. None		
	3. None		
Chapman 2013 [56]	1. None		
	2. In the general trauma population, LY30 of greater than 3% was associated with MT in 16.7% of the patients vs. 2.1% of those with LY30 < 3% (*p* = .006).		
	3. Similarly, LY30 ≥ 3% was associated with all-cause mortality of 20.8% vs. 4.7% (*p* = .011).		
Harr 2013 [44]	1. Functional Fibrinogen Levels (FF) significantly correlated with von Clauss fibrinogen levels (R^2^ = 0.87) and MA (R^2^ = 0.80). The mean fibrinogen contribution to MA was 30%; however, there was a direct linear relationship with fibrinogen level and% fibrinogen contribution to MA (R^2^ = 0.83). The addition of fibrinogen concentrate in *in vitro* studies increased MA (60.44 ± 1.48 to 68.12 ± 1.39) and % fibrinogen contribution to MA (23.8 ± 1.8% to 37.7 ± 2.5%).		
	2. None		
	3. None		
Johansson 2013 [45]	1. TEG® FF MA and G were lower in the hypocoagulable and significantly higher in hypercoagulable patients compared to patients with normal kaolin TEG® MA. By r-TEG®, R time, angle, MA, and G were reduced in hypocoagulable patients. LY30 was significantly increased in hypocoagulable patients by both TEG® and r-TEG®		
	2. Of the investigated TEG®, FF, and r-TEG® variables, MA, G, and LY30 were univariate predictors of MT whereas none were independent predictors of MT at 6 or 24 h		
	3. Nonsurvivors had significantly lower TEG® MA and lower FF MA and G compared to survivors. Further, r-TEG® angle and LY30 were lower in nonsurvivors.		
Lee 2013 [46]	1. There was a strong correlation between the r-TEG® and TEG® MA, which represents platelet function (R = .80). There was a moderate correlation between the G (R = .70) the overall clot strength, k (R = .66) speed of clot formation, and α-angle (R = .38), which reflects the degree of fibrin cross-linking. Lysis at 30 minutes correlated poorly (R = .19).		
	2. None		
	3. None		
Tapia 2013 [63]	1. None		
	2. None		
	3. TEG®-directed resuscitation is superior to MTP in MT penetrating trauma receiving ≥10U RBC. TEG®-directed resuscitation is equivalent to standardized MTP for all patients receiving ≥6U RBC and is also equivalent to standardized MTP for blunt trauma receiving ≥10U RBC. MTP worsened mortality in penetrating trauma receiving ≥10U RBC, indicating a continued need for TEG®-directed therapy.		
Kornblith 2014 [47]	1. Coagulopathic patients (INR ≥ 1.3) had lower admission MA FF than non-coagulopathic patients (24.7% vs. 31.2%, *p* < .05). %MA PLT was higher than MA FF at all-time points, decreased over time, and stabilized at 72 h (69.4% at 0 h, 56.2% at 72 h). In contrast, MA FF increased over time and stabilized at 72 hours (30.6% at 0 h, 43.8% at 72 h).		
	2. Patients requiring FFP had a significantly lower admission MA FF (26.6% vs. 30.6%, *p <* .05).		
	3. Higher admission MA FF was predictive of reduced mortality (hazard ratio, 0.815, *p* < .001).		
Branco 2014 [48]	1. 26.3% were hypercoagulable, 55.9% had a normal TEG® profile, and 17.8% were hypocoagulable.		
	2. After adjustment, hypercoagulable patients were less likely to require uncross-matched blood (adjusted *p* = .004) and less total blood products, in particular, plasma at 6 h (adjusted *p* < .001) and 24 h (adjusted *p* < .001).		
	3. Hypercoagulable patients had lower 24 h mortality (0.0% vs. 5.5% vs. 27.8%, adjusted *p <* .001) and 7-day mortality (0.0% vs. 5.5% vs. 36.1%, adjusted *p <* .001). Bleeding-related deaths were less likely in the hypercoagulable group (0.0% vs. 1.8% vs. 25.0%, adjusted *p <* .001).		

Table legend: A10 – clot amplitude at 10 minutes, A15 – clot amplitude at 15 minutes, AA – arachidonic acid, ACT – activated clotting time, ADP – adenosine diphosphate, ANOVA – analysis of variance, α angle – rate of clot formation, aPTT – activated partial thromboplastin time, AT – antithrombin, ATC – acute trauma coagulopathy, AUC – are under the curve, BD – base deficit, BE – base excess, BP – blood pressure, CFT – clot formation time, CI – confidence interval, CLI – clot lysis index, CT – clotting time, ED – emergency department, ELT – euglobin lysis time, EXTEM – extrinsically-activated test with tissue factor, F 1 + 2 – prothrombin fragment 1 + 2, FF – functional fibrinogen test, FFP – fresh frozen plasma, FIBTEM – fibrin-based extrinsically activated test with tissue factor and the platelet inhibitor cytochalasin D, G – shear elastic modulus strength ([5000 – MCF] / [100 – MCF] in ROTEM® and [5000 – MA] / [100 – MA] in TEG®), HCR – hemostatic control resuscitation, HF – hyperfibrinolysis, INTEM – intrinsically-activated test using ellagic acid, INR – international normalized ratio, ISS – injury severity score, K – kinetic time (time between 2 and 20 mm amplitude achieved in TEG®), LR – logistic regression, LY30 – percent decrease in clot amplitude at 30 min after MA in TEG®, MA – maximal amplitude, MCF – maximal clot firmness, ML – maximum lysis, MODS – multiple organ dysfunction syndrome, MRTG – maximum rate of thrombin formation, MT – massive transfusion, NPV – negative predictive value, OR – operating room, PE – pulmonary embolism, PCC – prothrombin complex concentrate, PF – primary fibrinolysis, PLT – platelet concentrate, PM – platelet mapping, PPV – positive predictive value, PT – prothrombin time, R – mean time for clot formation, RBC – red blood cells, RISC – revised injury severity classification, ROC – receiver operating curve, RSCT – routine screening coagulation tests, RTS – revised trauma score, SBP – systolic blood pressure, TAT – thrombin antithrombin complex, TBI – traumatic brain injury, TE – thromboembolic event, TEG®-PM – TEG® platelet mapping, TEM – thromboelastometry, TNF-α – tumor necrosis factor alpha, t-PA – tissue plasminogen activator, TRISS – trauma injury severity score.

#### Diagnosis of early trauma coagulopathies

##### Hypercoagulability

Six studies demonstrated hypercoagulability in trauma patients not detected by RSCT. Hypercoagulability was defined mostly by the manufacturer of both TEG® and ROTEM® devices, and the reference standards (where reported) were Doppler ultrasound, CT angiography, or a surgical procedure demonstrating thrombus. One study [11] demonstrated that 62% of trauma patients were hypercoagulable according to TEG® (R <3.7) on the first day after injury with normal RSCT values. Another study [19] showed higher TEG® α-angle (which reflects the degree of fibrin cross-linking) and MA (maximal amplitude) in trauma compared with RSCT (PT and aPTT) (*P* <0.05), indicating a hypercoagulable state. A study [24] detected increased *G* (shear elastic modulus strength (5,000 – MA)/(100 – MA), which reflects clot strength) in a cohort of trauma patients after splenectomy, who had more thromboembolic events, compared with patients treated nonoperatively. Another study of rapid TEG® (r-TEG®) [18] showed that increased *G* was associated with thromboembolic complications (OR, 1.25; 95% CI, 1.12 to 1.39), after controlling for thromboprophylaxis and using the reference standards discussed earlier. When TEG® [27] was performed 24 hours after injury, trauma patients were more hypercoagulable compared with healthy volunteers across a broad range of temperatures (32°C to 38°C).

Finally, a cohort study [34] suggested an association between admission MA and pulmonary embolism (OR, 3.5 for MA >65; 95% CI, 1.69 to 7.23; and OR, 5.8 for MA >72; 95% CI, 2.85 to 11.77), after controlling for gender, race, age, and ISS.

##### Hyperfibrinolysis (HF)

Only one study [14] compared HF detected with ROTEM® with a laboratory gold standard and showed that ROTEM® had satisfactory diagnostic properties for HF, defined by laboratory measurement of euglobin lysis time (ELT). However, the sample size was very small (*n* = 23, of which five had HF), limiting the strength of inferences.

##### Platelet dysfunction

With TEG® platelet-mapping (PM) test, a study [13] showed that patients with TBI had more platelet dysfunction on admission, measured by lower platelet response to arachidonic acid (AA) but not to adenosine diphosphate (ADP), compared with non-TBI trauma patients, alcohol abusers, and healthy volunteers (*P* <0.001). Another study [41], using the same technique, found lower platelet response to both AA and ADP in trauma patients versus healthy volunteers (*P* <0.0001). With ROTEM®, one study [42], comparing healthy volunteers with trauma patients, speculated that an observed difference in clot strength arose from platelet dysfunction. A related study [65] of ROTEM® at emergency department (ED) admission demonstrated significantly lower values of platelet component of clot elasticity (MCE EXTEM, ROTEM® extrinsically activated test with TF and MCE FIBTEM, ROTEM® fibrin-based extrinsically activated test with TF and the platelet inhibitor cytochalasin D) in trauma nonsurvivors vs. survivors (*P* = 0.0012).

##### Hypocoagulability

Six studies directly compared TEG® or ROTEM® with RSCT, with variable results for diagnostic performance. One study [9] demonstrated that TEG® detected hypocoagulability in 45 (85.5%) of 52 patients, whereas only one (1.9%) of 52 was hypocoagulable by elevated PT/aPTT and two (3.8%) of 52 were hypercoagulable by elevated platelet count. In a cohort [12] of trauma patients and healthy volunteers, EXTEM A15 ≥ 32 mm (amplitude at 15 minutes) and FIBTEM A10 ≥ 5 mm were sensitive (87% and 91%) and specific (100% and 85%) for detection of PT >1.5 of control value and fibrinogen <1 g/L, respectively. Doran [20] found that 16 (64%) of 25 patients were hypocoagulable by ROTEM® trace, and only 10% had abnormal RSCT (*P* = 0.0005). In contrast, another study [39] (*n* = 219) found that TEG®-R (R, reaction time, defined as the time until a clot firmness of 2 mm is achieved, corresponding to CT, clotting time, in ROTEM®) performed worse than INR for the diagnosis of vitamin K deficiency in trauma patients (clotting factor activity used as gold standard). TEG®-R (compared with INR >1.5) had a sensitivity of 33% (67% for INR), specificity of 95% (98% for INR), positive predictive value (PPV) of 47% (84% for INR), and negative predictive value (NPV) of 92% (96% for INR). In another cohort [26] (*n* = 300), ROTEM® parameters of CFT (clot-formation time), α-angle, A5 (clot amplitude 5 minutes after CT) and MCF (maximum clot firmness) were significantly different in the group with coagulopathy, defined by INR >1.2. A5 ≤ 35 mm had a sensitivity of 77% and specificity of 87% for the detection of coagulopathy. Finally, a recent large study [40] (*n* = 517) found that ROTEM® EXTEM, and FIBTEM measures of A5 and MCF were significantly correlated with fibrinogen levels (EXTEM A5, r^2^ = 0.35, and MCF, r^2^ = 0.26; FIBTEM A5, r^2^ = 0.44, and MCF, r^2^ = 0.27; all *P* <0.001). The sensitivity/specificity of EXTEM A5 < 36 mm (FIBTEM A5 < 9.5 mm) for discriminating patients with admission fibrinogen <1.5 g/L were 53%/87% (78%/70%; ROC AUC 0.8, 95% CI 0.7 to 0.9 for both).

One study [46] comparing the same parameters measured by r-TEG® and conventional kaolin-activated TEG® found strong correlation for MA (marker of platelet function; *r* = 0.80); moderate correlation for G (overall clot strength; *r* = 0.70), k (speed of clot formation; *r* = 0.66), and α-angle (degree of fibrin cross-linking; *r* = 0.38); and poor correlation for LY30 (degree of fibrinolysis; *r* = 0.19).

Although TEG® and r-TEG® may be moderately sensitive in detecting abnormal clot strength, they have not differentiated between fibrinogen and platelet contributions to clot integrity. Recent studies of the TEG®-based functional fibrinogen assay (FF) have examined the relative contribution each. By using Kaolin TEG® MA to define coagulopathy, Johansson [45] showed that TEG®-FF MA and G were lower in hypocoagulable patients and significantly higher in hypercoagulable patients compared with patients with normal Kaolin TEG® MA (*P* <0.001). In another study [47], coagulopathic patients (INR ≥1.3) had lower admission fibrinogen contribution to MA than did noncoagulopathic patients (24.7% versus 31.2%; *P* <0.05). Platelet contribution to MA was higher than fibrinogen at all time points, decreased over time, and stabilized at 72 hours (69.4% at 0 hours, 56.2% at 72 hours). In contrast, fibrinogen contribution to MA increased over time and stabilized at 72 hours (30.6% at 0 hours, 43.8% at 72 hours).

#### Turnaround times

Four studies reported on the use of TEG® and ROTEM® as POC devices. Carroll [16] demonstrated no statistical difference in the diagnosis of acute trauma coagulopathies when collecting blood on site or 1 hour after admission in the emergency department (ED), except for a small but statistically significant change in MA (60.6 (SD 11.1) mm on site and 63.4 (SD 12.1) mm in ED; *P* = 0.014). A cohort study [17] demonstrated that r-TEG® had a shorter TAT (time to MA) by a median (IQR) of 10.8 (1.1 to 18.5) minutes compared with TEG®. In another cohort [26], laboratory PT had a median TAT of 78 minutes (IQR, 62 to 103 minutes), whereas ROTEM® A5 was available by 5 minutes.

Finally, in another study [25], early r-TEG® values (ACT [activated clotting time], *k*-time, and *r*-value) were available within 5 minutes, late r-TEG® values (MA and α-angle) within 15 minutes, and RSCTs within 48 minutes (*P* <0.001 for all comparisons with r-TEG®).

#### Blood transfusion

##### Prediction of massive transfusion (MT) and any transfusion

Several studies compared ROTEM® and TEG® parameters with RSCTs for prediction of MT (defined by most studies as transfusion of ≥10 RBC units within 24 hours of trauma). Davenport [26] found better sensitivity (71% versus 42%) for ROTEM® A5 ≤ 35 mm versus INR >1.2, but worse specificity (85% versus 94%). Another study [31] found that FIBTEM (0 to 3 mm) had the highest AUC (0.84; 95% CI, 0.79 to 0.88) among ROTEM® tests for prediction of MT, but hemoglobin (AUC 0.87; 95% CI, 0.83 to 0.91) and PT (AUC, 0.87; 95% CI, 0.83 to 0.90) were better predictors.

A third study [22] showed multiple ROTEM® tests to be associated with MT; in a multivariable analysis limited by few events, hemoglobin ≤100 g/L (OR, 18.18; 95% CI, 2.73 to 125.00) was a stronger predictor of MT than abnormal MCF (OR, 8.47; 95% CI, 1.19 to 62.50). Similarly, RCSTs and TEG® had similar abilities to predict MT (G AUC 0.89; 95% CI, 0.89 to 0.96; INR AUC, 0.92; 95% CI, 0.86 to 0.98; PTT AUC 0.90; 95% CI, 0.83 to 0.97) [54].

ROTEM® parameters significantly associated with MT include increased CFT [22,31], decreased MCF [22,31], prolonged EXTEM and INTEM CT (intrinsically activated test using ellagic acid, clotting time) [31] and FIBTEM A10 (ROC AUC, 0.83; 95% CI, 0.78 to 0.87) [31]. For TEG®, statistically significant differences in α angle, MA, K, G (at 1 hour), and estimated lysis according to transfusion need (minimal, moderate, or massive) were reported [21]. Another study [36] found that patients with HF, defined by estimated lysis >15%, had a greater need for MT (76.9% versus 8.7%; adjusted OR, 19.1; 95% CI, 3.6 to 101.3).

Finally, a recent study demonstrated that a TEG® LY30 (percentage decrease in clot amplitude at 30 minutes after MA) of 3% or greater had poor sensitivity (31%) but high specificity (91%) for predicting MT [56].

Investigators have also compared TEG®/ROTEM® with RCST for prediction of any blood-product transfusion, generally guided by RSCT or a massive transfusion protocol (MTP). One study [52] found that r-TEG® *α*-angle <74.7 degrees had higher sensitivity (84%) but lower specificity (57%) to predict transfusion of any blood product versus INR >1.2 (38%, 88%), INR >1.5 (19%, 96%), or aPTT >60 (5%, 98%); fibrinogen <3 g/L had the highest sensitivity (90%) and similar specificity (48%). Another study [26] showed a similar pattern for ROTEM® A5 ≤ 35 mm in prediction of any RBC (sensitivity 33%, specificity 88%) or FFP (36%, 87%) compared with INR >1.2 (17%, 96% for RBC; 21%, 96% for FFP).

Considered in isolation, many TEG®/ROTEM® abnormalities have been associated with transfusion of specific blood products. Reduced TEG® MA was associated with transfusion of RBC [15,29], FFP [29], and PLT [29]; TEG® ACT predicted RBC, FFP, and PLT transfusions within the first 2 hours [25]; *α*-angle <56 predicted MT of RBC, FFP, PLT, and cryoprecipitate [35]; and patients with r-TEG®-defined maximum rate of thrombin generation (MRTG) ≥9.2 mm/min at 3 hours received significantly less RBC, FFP, and cryoprecipitate in the first 6 hours [37]. A study combining several markers [53] showed that patients considered coagulopathic based on TEG® (R, K, *α-*angle, and MA) received more RBC (10 versus 0), FFP (7 versus 0), and PLT (3 versus 0) in the first 24 hours (*P* <0.05). More recently, the inhibition of ADP function was correlated with transfusion in the first 6 hours (59.6% inhibition (0 RBC) versus 96.1% inhibition (>1 RBC), *P* = .025) [41], and TEG®-defined hypocoagulable patients required more uncross-matched blood (adjusted *P* = 0.004) and FFP (adjusted *P* <0.001) at 24 hours compared with normocoagulable and hypercoagulable patients [48].

Finally, patients requiring FFP had a significantly lower admission fibrinogen contribution to MA by the FF assay (26.6% versus 30.6%; *P <*0.05). In contrast, one study [16] showed no significant differences in TEG® parameters (R, K, α-angle, MA, LY60 (percentage decrease in clot amplitude at 60 minutes after MA) in patients transfused with RBC, FFP, or PLT versus not; only MA-ADP, a measure of ADP-platelet activation, correlated with transfusion of any blood product (*P* = 0.004).

Similarly, nonsurviving patients with isolated TBI had more ROTEM®-defined coagulation abnormalities and received more RBCs (*P* = 0.016), FC (*P* = 0.01) and PCC (*P* <0.001) than survivors [23], and higher FIBTEM MCF was associated with decreased RBC transfusion (adjusted OR, 0.92 per 1-unit increase, 95% CI, 0.87 to 0.98) [32]. Patients with HF [55], based on plasmin-antiplasmin complex levels and ROTEM® maximum lysis (ML) >15%, required more transfusions of RBC, FFP, PLT, and cryoprecipitate (*P* <0.05 for all comparisons).

Few data exist on TEG®/ROTEM® to monitor coagulopathy in response to transfusion. Rourke *et al.* [40] showed that administration of high doses of FC (6 to 12 g) or cryoprecipitate (30 U) restored EXTEM and FIBTEM A5 and MCF to the level of patients with minor injuries; fibrinogen levels were maintained but not augmented after FC administration. Changes in ROTEM®-determined CT, A5, and MCF after the transfusion of 4 units of RBC, dependent on the RBC/FFP ratio (≥1:1, 1:2 to 3:4, <1:2) [50].

##### TEG®/ROTEM®-guided transfusions versus conventional guidance

A modeling study [49] (*n* = 44) suggested that transfusion guided by r-TEG® versus RSCT would reduce the proportion of patients needing blood-product transfusion from 73.1% to 53.9% (*P* = 0.03), driven by reductions in FFP administration (61.5% with INR trigger versus 26.9% with r-TEG®-ACT trigger, *P* = 0.003). No difference was predicted for transfusion of PLT (*P* = 0.32) or cryoprecipitate (*P* = 0.18). A cohort study reported lower exposure to blood products in 80 patients with ROTEM®-guided FC and PCC compared with a historical group (*n* = 601) for whom FFP and PLT transfusions were guided by clinical decision (generally RSCT) [51]. Transfusion of RBC (PLT) was avoided in 29% (91%) of patients in the ROTEM®-guided FC and PCC group compared with 3% (56%) in the RSCT-guided FFP and PLT groups (*P* <0.001).

#### Mortality

##### Prediction

TEG® and ROTEM® parameters have been compared with RCSTs for prediction of mortality. For prediction of coagulopathy-related death, G had a similar adjusted AUC (0.93, 95% CI, 0.87 to 0.98) compared with INR (AUC, 0.88; 95% CI, 0.80 to 0.97; *P* = 0.11) and aPTT (AUC, 0.89; 95% CI, 0.81 to 0.97; *P* = 0.19) [54]. In another analysis, r-TEG® G, MRTG, and total thrombin generation (TG) (*P* = 0.03 for each) discriminated between survivors and nonsurvivors, in contrast to INR at 6 hours (*P* = 0.10) [37]. Another study found that TEG®-defined hypercoagulable patients had lower 24-hour mortality (0 versus 5.5% (normocoagulable) versus 27.8% (hypocoagulable), 10 deaths total, adjusted *P <*0.001) and hospital mortality (11.1% versus 5.5% versus 38.9%, 20 deaths total, adjusted *P <*0.001) [48]. With ROTEM®, a study [23] showed similar discrimination for mortality in TBI by using EXTEM with cytochalasin D (FIBTEM) MCF (AUC, 0.77; 95% CI, 0.66 to 0.85) and aPTT (AUC, 0.79; 95% CI, 0.69 to 0.87). A second study (*n* = 334; 26 early deaths) [32] using RSCTs, ROTEM®, and clinical judgment to guide transfusion, demonstrated significant correlations among PT, aPTT, fibrinogen, platelet count, and ROTEM® measurements (all |Spearman *r*| >0.5). In separate logistic regression analyses, each adjusted for hemoglobin and base excess, PT, aPTT, CT, CFT, MCF, LI (all EXTEM) and FIBTEM MCF were associated with 24-hour mortality. However, the predictive abilities of ROTEM® measurements and RSCTs were not directly compared.

Considering TEG®/ROTEM® measurements alone, weak clot strength [16,21,29,32,37,45,47,54,57,58,61,62] and HF [14,16,21,23,32,33,35,36,38,40,45,55,56,60] have been associated with morbidity and mortality. For example, ROTEM®-defined HF was associated with multiorgan dysfunction syndrome (MODS) (*n* = 115; 63.2% in patients with HF versus 24.6% in patients without HF, *P* = 0.004) [38] and hospital mortality (*n* = 115; 52.2% versus 12.9%, *P* <0.001) [38]; five of five versus nine of 82, *P* <0.05 [14]). Similarly in another study [36], TEG®-defined HF (estimated percentage lysis >15%) was associated with increased 24-hour mortality (69.2% versus 1.9% without HF; adjusted OR, 55.8; 95% CI, 7.2 to 432.3), but the number of deaths (*n* = 12) was extremely small. Holcomb [35] showed that r-TEG® LY-30, along with most other r-TEG® parameters, was associated with mortality; the logistic regression models were not described. More recently, a study [47] examined associations of the FF assay with mortality and demonstrated that a higher admission contribution of FF to MA predicted lower mortality (unadjusted hazard ratio, 0.815; *P* <0.001; 95% CI and adjusted model not reported).

##### Effect of TEG®/ROTEM®-guided transfusion

Several observational studies have examined whether TEG®/ROTEM®-guided transfusion reduced mortality after trauma and found no consistent effect. A small before/after study (*n* = 68) found lower crude mortality in patients in whom resuscitation was guided by r-TEG® (29% versus 65%) but did not report statistical testing or an adjusted analysis [37]. In a retrospective cohort study of massively bleeding patients (*n* = 131) transfused FC, PLT, and PCC using ROTEM® guidance, the observed mortality was 24.4%, which was lower than the expected mortality by TRISS (33.7%; *P* = 0.032) but similar to expected mortality by RISC (28.7%, *P >*0.05) [59]. Similarly, another cohort study of trauma patients (*n* = 681) found no difference in mortality between those treated in one center with ROTEM®-guided administration of FC and PCC (7.5%) and a multicenter control group treated with plasma transfusion (10%, *P* = 0.69) guided by the usual RSCT-guided clinical practice [51]. A before/after study [63] (*n* = 289) compared outcomes in trauma patients transfused with at least 6 U RBC in the first 24 hours according to TEG®-driven practice to a later period when a MTP not guided by TEG® was used. Overall, unadjusted mortality was unchanged, and MTP versus TEG®-directed care was not associated with mortality in multivariable analysis.

## Discussion

### Main findings

Our systematic review found 55 studies of TEG®/ROTEM® examining the diagnosis of trauma coagulopathies, including hypocoagulation, hypercoagulation, platelet dysfunction and fibrinolysis; guidance of blood-product administration; and associations with mortality. To our knowledge, this review is the first to summarize the literature on the use of TEG® and ROTEM® in trauma. The overall methodologic quality of included studies was moderate. No RCTs were reported; most cohort studies lacked clinically similar control groups managed without TEG®/ROTEM®, and standard measures of diagnostic accuracy were inconsistently reported. Observational data suggest that TEG® and ROTEM® may have adequate diagnostic properties for abnormalities identified by RSCTs and may identify additional coagulation disorders. However, the effect of these tests on the need for blood-product transfusion and mortality is unclear.

Studies in different but related clinical settings, not included in this systematic review, have also investigated TEG® and ROTEM®. Two studies of ROTEM® in a mixed population with shock [66] and noncardiac surgery [67] demonstrated rapid and useful results to guide decisions in hemostatic resuscitation. Other studies with mixed trauma and nontrauma populations [68-71] demonstrated good ability of ROTEM® to predict the need for MT, a clinically useful outcome regardless of management approach (laboratory abnormality-directed versus blood-product ratio based). Another possible advantage of these tests is that timely results may avoid FFP transfusion and subsequent FFP-related adverse events. However, relevant RCTs to test these hypotheses are lacking.

Other randomized [72-74] and observational [75-77] studies in cardiac surgery, burns, and mixed perioperative settings found reduced blood product transfusion and improved clinical outcomes after implementation of POC coagulation-management algorithms guided by ROTEM®. In contrast, existing observational studies in trauma patients do not suggest an effect of TEG®/ROTEM®-based transfusion protocols on clinically important outcomes, including mortality.

### Strengths and weaknesses of this study

Major limitations of this review are related to the quality of the included studies. Reproducible technical standards for the performance of TEG®/ROTEM® were lacking, and inconsistent reporting of 2 × 2 tables precluded calculation of summary diagnostic test-performance measures and exploration of threshold effects. A major problem faced by diagnostic studies of trauma coagulopathy is the ambiguous nature of the gold standard, given that RSCTs may not provide an adequate description of all associated abnormalities. No RCTs exist in trauma patients, aside from one that enrolled burns patients exclusively [73], and the quality of the observational studies is modest. Studies differed in the use of TEG® or ROTEM®, and the few studies [78,79] that compared TEG® and ROTEM® concluded that these methods are not interchangeable.

Studies also examined different patient populations, transfusion triggers, and transfusion protocols, limiting direct comparisons and generalizability. Clinical differences between many included studies and contemporary practice include substitution of FFP for clotting factors concentrate such as PCC, FC, and cryoprecipitate. Other methods for analysis of platelet dysfunction have been developed, such as platelet function analyzer (PFA-100) and multiple platelet function analyzer (Multiplate). These analyzers monitor different aspects of platelet function and appear to be technically reliable and practical POC devices, despite limitations [65,80-82].

Two systematic reviews of TEG®/ROTEM® exist for nontrauma populations. A Cochrane review [83] included nine RCTs, mostly in cardiac surgery, that compared transfusion guided by TEG®/ROTEM® with transfusion guided by clinical judgment, RSCTs, or both in severely bleeding patients. The review found that TEG®/ROTEM® reduced blood loss by a mean of 85 ml (95% CI, 29 to 141 ml) but had no effect on mortality.

Another systematic review [84] included 16 observational studies and two RCTs in patients with sepsis and concluded that TEG®/ROTEM® (compared with RSCTs) detect impaired fibrinolysis, which may help to discriminate between sepsis and systemic inflammatory response syndrome (SIRS). However, limitations of data prevented conclusions regarding the value of TEG®/ROTEM® to identify patients with sepsis who could benefit from anticoagulants.

## Conclusions

In summary, our systematic review demonstrated limited but rapidly growing observational evidence on the use of TEG® and ROTEM® in trauma. Both methods may be useful for diagnosis of early trauma coagulopathies, specifically hypocoagulability, hypercoagulability, hyperfibrinolysis, and platelet dysfunction. They may also be used to direct blood and blood-product transfusion; effects on patient-important outcomes are uncertain. The existing literature helps clinicians to appreciate the potential impact of these novel methods on transfusion guidance and outcomes in trauma. However, adequately powered and methodologically sound RCTs will be required to prove positive effects on blood-product transfusion and patient-important outcomes.

## Key messages

- The literature on TEG® and ROTEM® in trauma is limited by the lack of randomized controlled trials and the moderate quality of observational studies.
- TEG® and ROTEM® may be superior to routine screening coagulation tests to promptly diagnose early trauma coagulopathy, including hypocoagulability, hyperfibrinolysis, hypercoagulability, and platelet dysfunction.
- Many TEG® and ROTEM® abnormalities predict the need for massive transfusion and predict death, but predictive performance is not consistently superior to routine screening coagulation tests.
- Limited evidence from one observational study suggests that a ROTEM®-based transfusion algorithm reduces the amount of blood and blood products transfused.
- TEG® and ROTEM®-based resuscitation for bleeding trauma patients is not associated with lower mortality in most observational studies, but the question requires evaluation in randomized trials.

## Additional file

Additional file 1
**Details of literature search methods and references for the excluded studies.**

## Abbreviations

A10: Clot amplitude at 10 minutes
A15: clot amplitude at 15 minutes
A5: clot amplitude at 5 minutes after CT (in ROTEM®)
AA: arachidonic acid
ACT: activated clotting time
ADP: adenosine diphosphate
aPTT: activated partial thromboplastin time
AT: antithrombin
ATC: acute trauma coagulopathy
AUC: area under the curve
BD: base deficit
BE: base excess
BP: blood pressure
CFT: clot-formation time
CI: confidence interval
CLI: clot lysis index (residual clot firmness in percentage of MCF at a certain time after CT)
CT: clotting time
ED: emergency department
ELT: euglobulin lysis time
EXTEM: extrinsically activated test with tissue factor
F 1 + 2: prothrombin fragment 1 + 2
FF: functional fibrinogen test
FFP: fresh frozen plasma
FIBTEM: fibrin-based extrinsically activated test with tissue factor and the platelet inhibitor cytochalasin D
G: shear elastic modulus strength ([5,000 – MA]/[100 – MA])
HCR: hemostatic control resuscitation
HF: hyperfibrinolysis
INR: international normalized ratio
INTEM: intrinsically activated test using ellagic acid
IQR: interquartile range
ISS: injury severity score
K: kinetic time (time between 2 and 20 mm amplitude achieved in TEG®)
LI30: lysis index (residual clot firmness in percentage of MCF) 30 minutes after CT in ROTEM®)
LI60: lysis index (residual clot firmness in percentage of MCF) 60 minutes after CT in ROTEM®)
LR: logistic regression
LY30: percentage decrease in clot amplitude at 30 minutes after MA in TEG®
LY60: percentage decrease in clot amplitude at 60 minutes after MA in TEG®
MA: maximal amplitude
MCF: maximal clot firmness
ML: maximal lysis
MODS: multiple organ-dysfunction syndrome
MRTG: maximal rate of thrombin formation
MT: massive transfusion
NOS: Newcastle-Ottawa scale
NPV: negative predictive value
OR: odds ratio
PCC: prothrombin complex concentrate
PE: pulmonary embolism
PF: primary fibrinolysis
PFA-100: platelet-function analyzer
PLT: platelet concentrate
PM: platelet mapping
POC: point-of-care
PPV: positive predictive value
PRISMA: preferred reporting items for systematic reviews and meta-analyses
PT: prothrombin time
QUADAS: quality assessment of diagnostic accuracy studies
R: reaction time (time from starting the test until 2-mm amplitude can be detected in TEG®)
RBC: red blood cell
RCT: randomized controlled trial
RISC: revised injury severity classification
ROC: receiver operating curve
ROTEM®: rotational thromboelastometry
RSCT: routine screening coagulation test
r-TEG®: rapid thromboelastography
RTS: revised trauma score
SBP: systolic blood pressure
SD: standard deviation
SIRS: systemic inflammatory response syndrome
TAT: thrombin-antithrombin complex
TBI: traumatic brain injury
TE: thromboembolic event
TEG®: thromboelastography
TEG®-PM: TEG® platelet mapping
TF: tissue factor
TG: thrombin generation
TNF-α: tumor necrosis factor alpha
TRISS: trauma injury severity score
α angle: rate of clot formation
