# Supplementary material for: Effect of thromboelastography (TEG®) and rotational thromboelastometry (ROTEM®) on diagnosis of coagulopathy, transfusion guidance and mortality in trauma: descriptive systematic review
Source: Crit Care. 2014 Sep 27;18(5):518. doi: 10.1186/s13054-014-0518-9 (PMC4206701; doi:10.1186/s13054-014-0518-9)
Supplement: Additional file 1 — Details of literature search methods and references for the excluded studies. [file 13054_2014_518_MOESM1_ESM.pdf]

Additional file 1. Effect of thromboelastography (TEG®) and rotational thromboelastometry (ROTEM®) on diagnosis of coagulopathy, transfusion guidance and mortality in trauma: descriptive systematic review

This file provides details of the search strategy for this systematic review and provides a list of the excluded studies and the reason they were excluded from the qualitative analysis.

1. Data Sources and Searches

Database: Ovid MEDLINE(R) and Ovid OLDMEDLINE(R)
<1946 to February 2014>
Search Strategy:
1 Thrombelastography/ (3433)
2 (thromboelastography or thromboelastometry or TEG or ROTEM).mp. (2103)
3 exp "Wounds and Injuries"/ (700131)
4 (trauma* or injury or injuries).mp. (839498)
5 (1 or 2) and (3 or 4) (405)

Database: Embase Classic+Embase
<1947 to February 2014>
Search Strategy:
1 Thromboelastography/ (5583)
2 (thromboelastography or thromboelastometry or TEG or ROTEM).mp. (7214)
3 exp injury/ (1668459)
4 (trauma* or injury or injuries).mp. (1321622)
5 (1 or 2) and (3 or 4) (1046)

Database: EBM Reviews - Cochrane Database of Systematic Reviews
<2005 to February 2014>
Search Strategy:
1 [Thromboelastography/] (0)
2 (thromboelastography or thromboelastometry or TEG or ROTEM).mp. (10)
3 [exp "Wounds and Injuries"/] (0)
4 (trauma* or injury or injuries).mp. (2793)
5 (1 or 2) and (3 or 4) (8)

Database: EBM Reviews - Cochrane Central Register of Controlled Trials <February 2014>
Search Strategy:
1 Thromboelastography/ (147)
2 (thromboelastography or thromboelastometry or TEG or ROTEM).mp. (180)

3 exp "Wounds and Injuries"/ (11796)
4 (trauma* or injury or injuries).mp. (21833)
5 (1 or 2) and (3 or 4) (8)

2. Studies excluded after full text evaluation

a. mixed population (trauma and other surgical or clinical bleeding settings)

1. Viljoen M, Roux LJ, Pretorius JP, Coetzee IH, Viljoen E: **Hemostatic competency and elastase-alpha 1-proteinase inhibitor levels in surgery, trauma, and sepsis.** *J Trauma* 1995, **39**(2):381-385.

2. Johansson PI, Stensballe J: **Effect of haemostatic control resuscitation on mortality in massively bleeding patients: a before and after study.** *Vox Sang* 2009, **96**(2):111-118.

3. Johansson PI, Bochsen L, Stensballe J, Secher NH: **Transfusion packages for massively bleeding patients: the effect on clot formation and stability as evaluated by Thrombelastograph (TEG).***Transfus Apher Sci* 2008, **39**(1):3-8.

4. Lancé MD, Ninivaggi M, Schols SE, Feijge MA, Oehrl SK, Kuiper GJ, Nikiforou M, Marcus MA, Hamulyak K, Van Pampus EC, Cate H, Heemskerk JW: **Perioperative dilutional coagulopathy treated with fresh frozen plasma and fibrinogen concentrate: a prospective randomized intervention trial.** *Vox Sang* 2012, **103**(1):25-34.

5. Görlinger K, Dirkmann D, Solomon C, Hanke AA: **Fast interpretation of thromboelastometry in non-cardiac surgery: reliability in patients with hypo-, normo-, and hypercoagulability.** *Br J Anaesth* 2013, **110**(2):222-230.

6. Stravitz RT, Lisman T, Luketic VA, Sterling RK, Puri P, Fuchs M, Ibrahim A, Lee WM, Sanyal AJ: **Minimal effects of acute liver injury/acute liver failure on hemostasis as assessed by thromboelastography.** *J Hepatol* 2012, **56**(1):129-136.

7. Görlinger K, Fries D, Dirkmann D, Weber CF, Hanke AA, Schöchl H: **Reduction of fresh frozen plasma requirements by perioperative point-of-care coagulation management with early calculated goal-directed therapy.** *Transfus Med Hemother* 2012, **39**(2):104-113.

8. Harr JN, Moore EE, Chin TL, Ghasabyan A, Gonzalez E, Wohlauer MV, Banerjee A, Silliman CC, Sauaia A: **Platelets are dominant contributors to hypercoagulability after injury.** *J Trauma Acute Care Surg* 2013, **74**(3):756-762.

9. Theusinger OM, Schröder CM, Eismon J, Emmert MY, Seifert B, Spahn DR, Baulig W: **The influence of laboratory coagulation tests and clotting factor levels on Rotation Thromboelastometry (ROTEM(R)) during major surgery with hemorrhage.** *Anesth Analg* 2013, **117**(2):314-321.

10. Sawhney C, Subramanian A, Kaur M, Anjum A, Albert V, Soni KD, Kumar A: **Assessment of hemostatic changes after crystalloid and colloid fluid preloading in trauma patients using standard coagulation parameters and thromboelastography**. *Saudi J Anaesth* 2013, **7**(1):48-56.

11. Reed MJ, Nimmo AF, McGee D, Manson L, Neffendorf AE, Moir L, Donaldson LS: **Rotational thrombolelastometry produces potentially clinical useful results within 10 min in bleeding emergency department patients: the DEUCE study.** *Eur J Emerg Med* 2013, **20**(3):160-166.

12. McCully SP, Fabricant LJ, Kunio NR, Groat TL, Watson KM, Differding JA, Deloughery TG, Schreiber MA: **The international normalized ratio overestimates coagulopathy in stable trauma and surgical patients.** *J Trauma Acute Care Surg* 2013, **75**(6):947-953.

b. Non-traumatic brain bleeding

1. Windeløv NA, Welling KL, Ostrowski SR, Johansson PI: **The prognostic value of thrombelastography in identifying neurosurgical patients with worse prognosis.** *Blood Coagul Fibrinolysis* 2011, **22**(5):416-419

2. Frontera JA, Aledort L, Gordon E, Egorova N, Moyle H, Patel A, Bederson JB, Sehba F: **Early platelet activation, inflammation and acute brain injury after a subarachnoid hemorrhage: a pilot study.** *J Thromb Haemost* 2012, **10**(4):711-713.

c. Burns

1. Schaden E, Hoerburger D, Hacker S, Kraincuk P, Baron DM, Kozek-Langenecker S: **Fibrinogen function after severe burn injury.** *Burns* 2012, **38**(1):77-82.

2. Van Haren RM, Thorson CM, Valle EJ, Busko AM, Guarch GA, Andrews DM, Pizano LR, Schulman CI, Namias N, Proctor KG: **Hypercoagulability after burn injury.** *J Trauma Acute Care Surg* 2013, **75**(1):37-43.

d. Miscellaneous

1. Kashuk JL, Moore EE, Johnson JL, Biffl WL, Burlew CC, Barnett C, Sauaia A: **Progressive postinjury thrombocytosis is associated with thromboembolic complications.** *Surgery* 2010, **148**(4):667-74.

2. Dirks J, Jørgensen H, Jensen CH, Ostrowski SR, Johansson PI: **Blood product ratio in acute traumatic coagulopathy--effect on mortality in a Scandinavian level 1 trauma centre.** *Scand J Trauma Resusc Emerg Med* 2010, **18**:65.

3. Gonzalez E, Kashuk JL, Moore EE, Silliman CC**: Differentiation of enzymatic from platelet hypercoagulability using the novel thrombelastography parameter delta (delta).** *J Surg Res* 2010, **163**(1):96-101.

4. Martinaud C, Ausset S, Deshayes AV, Cauet A, Demazeau N, Sailliol A: **Use of freeze-dried plasma in French intensive care unit in Afghanistan.** *J Trauma* 2011, **71**(6):1761-1764.

5. James MF, Michell WL, Joubert IA, Nicol AJ, Navsaria PH, Gillespie RS: **Resuscitation with hydroxyethyl starch improves renal function and lactate clearance in penetrating trauma in a randomized controlled study: the FIRST trial (Fluids in Resuscitation of Severe Trauma).** *Br J Anaesth* 2011, **107**(5):693-702.

6. Cole E, Davenport R, De'Ath H, Manson J, Brockamp T, Brohi K: **Coagulation system changes associated with susceptibility to infection in trauma patients**. *J Trauma Acute Care Surg* 2013, **74**(1):51-57.

7. Sølbeck S, Windeløv NA, Bæk NH, Nielsen JD, Ostrowski SR, Johansson PI: **In-vitro comparison of free oscillation rheometry (ReoRox) and rotational thromboelastometry (ROTEM) in trauma patients upon hospital admission.** *Blood Coagul Fibrinolysis* 2012, **23**(8):688-692.

8. Schaden E, Kimberger O, Kraincuk P, Baron DM, Metnitz PG, Kozek-Langenecker S: **Perioperative treatment algorithm for bleeding burn patients reduces allogeneic blood product requirements.** *Br J Anaesth* 2012, **109**(3):376-381.

9. Hagemo JS, Næss PA, Johansson P, Windeløv NA, Cohen MJ, Røislien J, Brohi K, Heier HE, Hestnes M, Gaarder C: **Evaluation of TEG(®) and RoTEM(®) inter-changeability in trauma patients.** *Injury* 2013, **44**(5):600-605.

10. Cohen MJ, Kutcher M, Redick B, Nelson M, Call M, Knudson MM, Schreiber MA, Bulger EM, Muskat P, Alarcon LH, Myers JG, Rahbar MH, Brasel KJ, Phelan HA, del Junco DJ, Fox EE, Wade CE, Holcomb JB, Cotton BA, Matijevic N. PROMMTT Study Group: **Clinical and mechanistic drivers of acute traumatic coagulopathy.** *J Trauma Acute Care Surg* 2013, **75**(1 Suppl 1):S40-7.

11. Fabricant L, Kiraly L, Wiles C, Differding J, Underwood S, Deloughery T, Schreiber M: **Cryopreserved deglycerolized blood is safe and achieves superior tissue oxygenation compared with refrigerated red blood cells: a prospective randomized pilot study.** *J Trauma Acute Care Surg* 2013, **74**(2):371-376.
